# Body Composition in Cholangiocarcinoma Affects Immune Cell Populations in the Tumor and Normal Liver Parenchyma

**DOI:** 10.1016/j.jceh.2024.102460

**Published:** 2024-11-26

**Authors:** Guanwu Wang, Dong Liu, Tarick M. Al-Masri, Carlos C. Otto, Jens Siveke, Sven A. Lang, Tom F. Ulmer, Steven WM Olde Damink, Tom Luedde, Edgar Dahl, Ulf P. Neumann, Lara R. Heij, Jan Bednarsch

**Affiliations:** ∗ Department of Surgery and Transplantation, University Hospital RWTH Aachen, Aachen, Germany; † University of Applied Science Aachen, Aachen, Germany; ‡ Department of Surgery and Transplantation, University Hospital Essen, Essen, Germany; § Institute for Developmental Cancer Therapeutics, West German Cancer Center, University Hospital Essen, Essen, Germany; ‖ Department of Surgery, Maastricht University Medical Centre (MUMC), Maastricht, Netherlands; ¶ Department of Gastroenterology, Hepatology and Infectious Diseases, Heinrich Heine University Duesseldorf, Duesseldorf, Germany; # Institute of Pathology, University Hospital RWTH Aachen, Aachen, Germany; ∗∗ Institute of Pathology, University Hospital Essen, Essen, Germany; †† German Cancer Consortium (DKTK), partner site Essen, a partnership between German Cancer Research Center (DKFZ) and University Hospital Essen, Germany

**Keywords:** cholangiocellular carcinoma, immune cells, body composition, oncological outcome

## Abstract

**Background:**

Due to malnutrition and tumor cachexia, body composition (BC) is frequently altered and known to adversely affect short- and long-term results in patients with cholangiocarcinoma (CCA). Here, we explored immune cell populations in the tumor and liver of CCA patients with respect to BC.

**Methods:**

A cohort of 96 patients who underwent surgery for CCA was investigated by multiplexed immunofluorescence (MIF) techniques with computer-based analysis on whole-tissue slide scans to quantify and characterize immune cells in normal liver and tumor regions. BC was characterized by obesity, sarcopenia, myosteatosis, visceral obesity and sarcopenic obesity. Associations between BC and immune cell populations were determined by univariate and multivariable binary logistic regressions.

**Results:**

BC was frequently altered in intrahepatic CCA (iCCA, n = 48), with 47.9% of the patients showing obesity, 70.8% sarcopenia, 18.8% sarcopenic obesity, 58.3% myosteatosis and 54.2% visceral obesity as well as in perihilar CCA (pCCA, n = 48) with 45.8% of the patients showing obesity, 54.0 sarcopenia, 14.6% sarcopenic obesity, 47.9% myosteatosis and 56.3% visceral obesity. From an immune cell perspective, independent associations within the tumor compartment were observed for iCCA (myosteatosis: TIM-3+CD8+cells; obesity: PD-1+TIM-3+CD4+cells) and for pCCA (myosteatosis: PD-L2+CD68-cells and CD4+cells). Further, independent associations were observed within the normal liver parenchyma for iCCA (visceral obesity: PD-1+PD-L1+PD-L2+CD68+cells) and for pCCA (sarcopenia: CD68+cells and TIM-3+CD8+cells; visceral obesity: ICOS+-TIGIT+CD8+cells and sarcopenic obesity: PD-1+PD-L1+PD-L2+CD8+cells).

**Conclusion:**

This is the first systematic analysis of the association of BC and immune cells in cholangiocarcinoma showing a strong association between BC and distinct immune cell populations within the tumor itself as well as within the normal parenchyma.

Cholangiocarcinoma (CCA) is a malignant tumor arising from epithelial cells of the bile ducts. It is responsible for around 15% of all primary liver cancers and is the second most common type of primary liver cancer after hepatocellular carcinoma (HCC).^1^ Despite advancements in diagnosis and treatment of CCA, survival rates remain low. Surgical resection is still the only curative option, but most patients present with unresectable or metastatic disease. A gemcitabine–cisplatin combination is recommended as systemic therapy for patients with advanced or inoperable CCA, but its survival benefit is only a few months.^2^ The era of immunotherapies in CCA is just emerging.

CCA is a proliferative tumor with abundant desmoplastic stroma. The tumor microenvironment (TME) of CCA tumors contains numerous cancer-associated fibrous cells (CAFs), lymphoid cells, tumor-associated macrophages (TAMs), tumor-associated neutrophils (TANs), regulatory T lymphocytes (Tregs), and natural killer (NK) cells, which play a critical role in promoting CCA growth and dissemination.^3^^,^^4^ However, our knowledge of the microenvironment of CCA tumors is elusive, greatly limiting the optimization of immunotherapies. Current immunotherapies for cholangiocarcinoma are mainly checkpoint inhibitors and the combination of immune checkpoint inhibitors (ICIs) with an additional checkpoint inhibitor, a cytotoxic chemotherapy or targeted therapies. However, immunotherapy is only effective in a subset of CCA patients, underlining the importance of identifying those patients who might be most responsive.^4^

Body composition (BC) parameters, such as body mass index (BMI), skeletal muscle mass and total fat mass, have been reported to be associated with postoperative morbidity and prognosis in several cancers.^5^ Obesity has been associated with an increased risk of certain tumor progression through chronic inflammation and the environmental effects on metabolic function that may accelerate immune senescence or failure.^6^ Due to cytokine-mediated inflammation coupled with malnutrition, cancer patients are at increased risk of developing sarcopenia, which may lead to loss of muscle mass and strength in cancer patients.^7^ Sarcopenia might also affect the inflammatory status and immune cells as it influences disease progression. For example, myosteatosis can predict unfavorable outcomes of anti-PD-1 immunotherapy in advanced hepatocellular carcinoma patients.^8^ Therefore, BC has been widely recognized as a prognostic parameter for solid tumor progression. However, so far, little is known about the relationship between BC alterations and immune cells in cholangiocarcinoma.

We hypothesized that BC may strongly affect immune composition and distribution and thereby influence the course of CCA. This study aims to examine the connection between BC and immune cells in patients with intrahepatic (iCCA) and perihilar CCA (pCCA) and to assess the prognostic significance of BC.

## MATERIALS AND METHODS

### Ethics approval and consent to participate

This study was conducted in accordance with the current version of the Declaration of Helsinki and good clinical practice guidelines (International Conference on Harmonization, Good Clinical Practice). Informed consent was obtained from the included patients. Approval was granted by the institutional review board (Reference: EK 341/21).

### Patients

The analysis included patients who underwent curative-intent surgery for iCCA (n = 48) and pCCA (n = 48) at a single hepatobiliary center between 2010 and 2019. During this time period, a total of 255 patients underwent surgery for either iCCA or pCCA. Excluding perioperative mortality, a subset of 226 patients was eligible for oncological analysis. Given the availability of tissue for multiple mIF experiments, the study cohort of 96 patients was applicable for this study. Prior to surgery, all patients underwent a thorough evaluation to rule out distant metastases and to assess the severity of hepatic and hilar disease as previously described.^9^ Formalin-fixed paraffin-embedded (FFPE) tissue blocks were selected from the patients. The study was conducted in accordance with the Declaration of Helsinki and the latest edition of the International Conference on Harmonization, Good Clinical Practice. This retrospective study was carried out according to the recommendations of the local ethics committee (EK 360/19).

### Computed Tomography Imaging and BC Analysis

All BC measurements were performed by two investigators (GW, DL) blinded to the clinical data of the patients. All computed tomography (CT) images underwent contrast enhancement, with the images being acquired in the venous phase. We performed semi-automated segmentation of skeletal muscle and adipose tissue in single cross-sectional CT images at the level of the third lumbar vertebra using the 3D Slicer software platform version 4.1 and the BC module (https://www.slicer.org/) (L3). Using attenuation values between -29 and 150 Hounsfield units (HU), the following skeletal muscles were assessed and quantified: quadratus lumborum, transversus abdominis, erector spinae, psoas major, external and internal obliques, and rectus abdominis. The skeletal muscle index (SMI) was calculated using the following formula: Total skeletal muscle cross-sectional area at L3/(height)2 (cm^2^/m^2^). Visceral fat area (VFA) was assessed using attenuation values between -150 and -50 HU, while subcutaneous fat area was obtained using attenuation values between -190 and 30 HU. HUs were evaluated for skeletal muscle radiation attenuation (SM-RA), an indicator of muscle density and myosteatosis.

The assessment of BC has historically used a variety of cutoff values.^5^ The definition of overweight or obesity was based on a body mass index (BMI) ≥ 25 kg/m^2^. Sarcopenia was diagnosed if the SMI was <41 cm^2^/m^2^ in women and <43 cm^2^/m^2^ in men with BMI <25 kg/m^2^, or if the SMI was <53 cm^2^/m^2^ with a BMI ≥25 kg/m^2^. Myosteatosis was diagnosed when SM-RA was <41 HU for BMI <25 kg/m^2^ or <33 HU for BMI ≥25 kg/m^2^. Visceral fat area (VFA) was considered high if ≥ 100 cm^2^. Sarcopenic obesity was diagnosed if SMI was ≤38.5 cm^2^/m^2^ for women and ≤52.4 cm^2^/m^2^ for men with BMI ≥25 kg/m^2^. Supplementary Figure 1 displays examples of all different BC characteristics.

### Sample Collection and Whole-slide Multiplexed Immunofluorescence

Hematoxylin and eosin (H&E) slides were collected and the slides with vital tumor and presence of normal liver tissue were selected by a dedicated gastrointestinal pathologist (LH). The selected block was used for further processing for our multiplexed imaging workflow using the TissueFAXS method (TissueGnostics, Austria). All FFPE samples were subjected to multiplexed immunofluorescence (mIF) in serial 5 μm histological tumor sections obtained from representative FFPE tumor blocks. The FFPE blocks were carefully selected with the presence of the tumor region and normal tissue. The sections were labeled by using the Opal 7-Color fIHC Kit (PerkinElmer, Waltham, MA). The antibody fluorophores were grouped into a panel of five antibodies as previously described.^10^ The order of antibodies staining was always kept constant on all sections and the sections were initially counterstained with DAPI (Vector Laboratories, Newark, USA). The multiplexed immunofluorescence panel consisted of CD4, CD8, CD68, PD-1, PD-L1, PD-L2, ICOS, TIGIT, TIM-3, CTLA-4, and LAG-3. All antibodies were diluted with antibody diluent (with background reducing components, Dako, Germany). Secondary antibodies were applied with ImmPRESS™ HRP (Peroxidase) Polymer Detection Kit (Vector Laboratories, US). TSA reagents were diluted with 1 × Plus Amplification Diluent (PerkinElmer/Akoya Biosciences, US).

The mIF was carried out in accordance to the Edwin R. Parra's protocol as previously described.^10^ The slides were then digitally scanned with the TissueFAXS PLUS system (TissueGnostics, Austria). Image analysis was performed in different regions of interest (ROI): tumor and normal liver. The size of the ROI varied per slide. Immune cell expression was calculated in percentages of the overall cell population in the ROI throughout the whole project as previously described.^11^

Strataquest software (TissueGnostics, Austria) was used to analyse the antibody staining and cell counts. The library information was used to associate each fluorochrome component with an mIF marker. All immune cell populations were quantified as positive cells per square mm using the cell segmentation, thresholds were set manually by a dedicated pathologist. Positive cell count was categorized based on thresholds, a value above the threshold was considered as positive as previously described.^11^

As described above, the number of positive cells for each marker was expressed as percentage of the overall cell count (DAPI) in the assessed ROI (normal liver or tumor). In case of co-expression, the cells were only counted as positive if each marker was assessed as positively stained. For statistical analysis, patients with iCCA and pCCA were separately assessed and grouped into high expression and low expression using the median percentage of positive cells referenced to the overall cell count.

### Statistical Analysis

Cancer-specific survival (CSS) was defined as the time from the date of surgery to cancer-associated death. Patients deceasing from other causes were censored at the time of death. Recurrence-free survival (RFS) is defined as the time from the date of surgery to the date of disease recurrence. The correlation among immune cell data, clinical variables, and BC was evaluated using univariate and multivariate logistic regression analyses. For this purpose, miF data were used to categorize iCCA and pCCA patients separately into a low and high expression group. Univariate and multivariate Cox regression analyses were performed to determine the association between clinical variables and RFS or CSS. The Kaplan–Meier method was used to construct survival curves. Multivariable models were built from variables displaying significance in univariate analysis (*P* < 0.05). To account for potential confounding variables, we included large dataset of variables into the univariate models including demographics, clinical chemistry, operative data, postoperative data, oncological characteristics as well as BC parameters and immune cell data. Additionally, iCCA and pCCA patients were analyzed separately due to their distinct biological and clinical profiles, which could otherwise introduce heterogeneity into the results. SPSS Statistics (version 26; IBM, Armonk, NY, USA) was used for all statistical analyses.

## RESULTS

### Characteristics of the Study Cohort

The study enrolled a total of 96 patients of which 48 were treated for iCCA (50.0%) and 48 for pCCA (50.0%). Patients with iCCA had a median age of 66 years and mostly underwent major liver resections (85.4%). After a median follow-up of 31 months, recurrence developed in 72.9% of the patients resulting in a median RFS of 8 months and a median cancer-specific survival (CCS) of 30 months (Supplementary Figure 2 A+B). Patients with pCCA had a median age of 67 years and also mostly underwent major liver resections (97.9%). After a median follow-up of 34 months, recurrence developed in 66.7% of the patients resulting in a median RFS of 29 months and a median CCS of 28 months (Supplementary Figure 2 C+D). More details are summarized in Table 1.

**Table 1:** Patient Characteristics.

Variables	iCCA（n = 48）	pCCA（n = 48）
**Demographics**		
Gender, M/F (%)	23 (47.9)/25 (52.1)	29 (60.4)/19 (39.6)
Age (years)	66 (56–73)	67 (56–73)
Portal vein embolization, n (%)	3 (6.3)	20 (41.7)
ASA, n (%)		
I	1 (2.1)	2 (4.2)
II	23 (47.9)	22 (45.8)
III	20 (41.7)	23 (47.9)
IV	4 (8.3)	1 (2.1)
V	0	0
Preoperative chemotherapy, n (%)	3 (6.3)	3 (6.3)
**Clinical chemistry**		

AST (U/l)	39.50 (26.00–54.75)	51.50 (37.25–94.00)
ALT (U/l)	27.00 (20.00–68.00)	84.50 (50.00–187.00)
GGT (U/l)	123.00 (68.25–401.75)	380.00 (207.50–830.00)
Total bilirubin (mg/dl)	0.55 (0.41–0.92)	1.10 (0.50–2.88)
Hemoglobin (g/dl)	13.20 (12.00–14.40)	12.10 (11.02–13.10)
Platelet count (/nl)	252 (197–326)	336 (250–420)
INR	1.00 (0.95–1.07)	1.04 (0.93–1.14)
Prothrombin time (%)	99.00 (88.50–106.50)	93.08 (80.00–109.00)
CRP (mg/l)	10.00 (10.00–27.6)	10.50 (5.0–35.5)
**Operative data**		
Operative time (minutes)	285 (230–354)	390 (351–4765)
Operative procedure, n (%)		
Atypical	3 (6.3)	0
Monosegmentectomy	0	0
Bisegmentectomy	4 (8.3)	0
Hemihepatectomy	18 (37.5)	12 (25.0)
Extended hemihepatectomy	7 (14.6)	17 (35.4)
Trisectionectomy	8 (16.7)	10 (20.8)
Hepatoduodenoectomy	0	8 (16.7)
ALPPS	8 (16.7)	1 (2.1)
Intraoperative PRBC, n (%)	20 (41.7)	26 (54.2)
Intraoperative FFP, n (%)	22 (45.8)	32 (66.7)
Intraoperative platelets, n (%)	1 (2.1)	0
**Pathological examination**		
R1 resection, n (%)	4 (8.3)	8 (16.7)
pN category, n (%)		
N0	31 (64.6)	22 (45.8)
N1	15 (31.3)	26 (54.2)
Tumor grading, n (%)		
G1	0	0
G2	35 (72.9)	36 (75.0)
G3	10 (20.8)	8 (16.7)
G4	0	1 (2.1)
MVI, n (%)	18 (37.5)	14 (29.2)
LVI, n (%)	9 (18.8)	12 (25.0)
pT category n (%)		
1	15 (31.3)	0 (0)
2	24 (50.0)	30 (62.5)
3	6 (12.5)	12 (25.0)
4	3 (6.3)	6 (12.5)
**Postoperative data**		
Intensive care, days	1 (1–2)	2 (1–3)
Hospitalization, days	14 (9–24)	23 (15–36)
Postoperative complications, n (%)		
No complications	16 (33.3)	6 (12.5)
Clavien–Dindo I	1 (2.1)	3 (6.3)
Clavien–Dindo II	14 (29.2)	11 (22.9)
Clavien–Dindo IIIa	6 (12.5)	10 (20.8)
Clavien–Dindo IIIb	7 (14.6)	13 (27.1)
Clavien–Dindo IVa	4 (8.3)	3 (6.3)
Clavien–Dindo IVb	0 (0)	2 (4.2)
Clavien–Dindo V	0 (0)	0 (0)
**Oncologic data**		
Adjuvant chemotherapy, n (%)	12 (25.0)	10 (20.8)
Recurrence, n (%)	35 (72.9)	32 (66.7)
Median RFS, months (95% CI)	8 (4–12)	29 (6–52)
Median CSS, months (95% CI)	30 (8–54)	28 (13–43)
**Body composition**		
BMI (kg/m^2^)	24.72 (22.55–29.05)	24.57 (22.01–26.58)
Visceral fat area (cm^2^)	118.24 (57.33–201.42)	119.66 (64.18–180.25)
SMI (cm^2^/m^2^)	41.93 (35.42–46.35)	43.73 (38.54–50.56)
Sarcopenia, n (%)	34 (68.0)	27 (54.0)
Myosteatosis, n (%)	28 (56.0)	23 (47.9)
Sarcopenic obesity, n (%)	9 (18.8)	7 (14.6)

Data presented as median and interquartile range if not noted otherwise.

ALPPS, associating liver partition with portal vein ligation for staged hepatectomy; ALT, alanine aminotransferase; ASA, American Society of Anesthesiology; AST, aspartate aminotransferase; BMI, Body mass index; CRP, C-reactive protein; CSS, cancer-specific survival; FFP, fresh frozen plasma; GGT, gamma-glutamyl transferase; iCCA, intrahepatic cholangiocarcinoma; INR, international normalized ratio; LVI, lymph vascular invasion; MVI, microvascular invasion; pCCA, Perihilar cholangiocarcinoma; PRBC, packed red blood cells; RFS, recurrence-free survival; SMI, skeletal muscle index.

### Association of Body Composition With Immune Cells in Intrahepatic Cholangiocarcinoma

Within the iCCA group, 23 (47.9%) patients were classified as obese, 34 (70.8%) showed sarcopenia, 9 (18.8%) displayed sarcopenic obesity, 28 (58.3%) showed myosteatosis and 26 (54.2%) displayed visceral obesity (Table 1). To associate these BC characteristics with clinical characteristics as well as immune cell composition, univariate und multivariable logistic regressions were conducted.

Here, patients with obesity showed a higher expression of CD4+PD-1+ (*P* = 0.027), CD4+PD-1+LAG3+TIM-3+ (*P* = 0.027), CD4+PD-1+TIM-3+ (*P* = 0.027), CD4+LAG3+TIM-3+ (*P* = 0.027), and CD8+PD-1+TIM-3+ (*P* = 0.027) cells within the tumor and were more likely to display lymph vascular invasion (LVI) (*P* = 0.037). In multivariable analysis, higher expression of CD4+PD-1+TIM-3+ (odds ratio [OR] = 8.904, *P* = 0.012) cells in the tumor tissue and LVI (OR = 14.990, *P* = 0.016) were the significant parameter associated with obesity.

**Figure 1:**
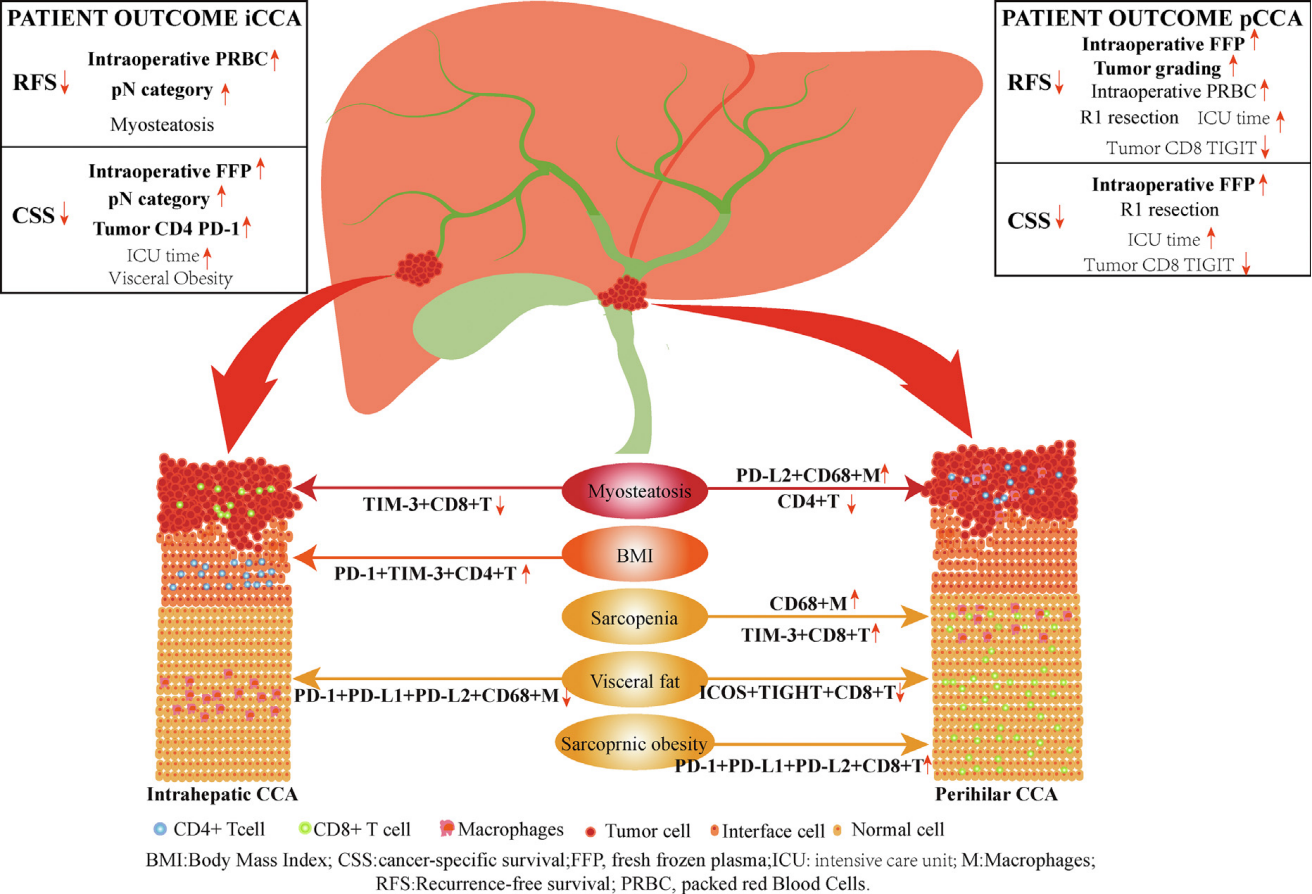
**Synopsis of body composition and immune cells**. Independent associations within the tumor compartment were observed for myosteatosis (iCCA: TIM-3+CD8+cells; pCCA: PD-L2+CD68-cells, CD4+cells), BMI (PD-1+TIM-3+CD4+cells) and within the normal liver parenchyma for sarcopenia (pCCA: CD68+cells, TIM-3+,CD8+cells), visceral obesity (iCCA: CD-1+PD-L1+PD-L2+CD68+cells; pCCA: ICOS+TIGIT+CD8+cells) and sarcopenic obesity (pCCA: PD-1+PD-L1+PD-L2+CD8+cells). Patient outcomes regarding RFS and CSS are also displayed. Variables being both significant in univariate and multivariate Cox regressions are indicated by bold letters, while variables being only significant in univariate analysis are displayed normal. CCS, cancer-specific survival; iCCA, intrahepatic cholangiocarcinoma; pCCA, perihilar cholangiocarcinoma; RFS, recurrence-free survival.

Individuals with sarcopenia were more likely female patients (OR = 4.038, *P* = 0.043) and did not show association with immune cell composition. In contrast, the presence of myosteatosis was associated with reduced expression of CD4+PD-1+LAG3+ (*P* = 0.024), CD8+PD-1+ (*P* = 0.024), CD8+LAG3+ (*P* = 0.024) and CD8+TIM-3+ (*P* = 0.005) cells within the tumor as well as reduced gamma-glutamyltransferase (GGT) (*P* = 0.013), reduced hemoglobin (*P* = 0.017) and female gender (*P* = 0.049). In multivariable analysis, patients with myosteatosis showed a lower CD8+TIM-3+ expression in tumor tissue (OR = 0.023, *P* = 0.118) and lower hemoglobin levels (OR = 0.104, *P* = 0.019). ICCA patients with visceral obesity displayed reduced CD68+PD1+ (*P* = 0.042) and CD68+PD1+PDL1+PDL2+ (*P* = 0.040) expression in the normal liver as well as higher hemoglobin levels (*P* = 0.048), shorter hospitalization (*P* = 0.042) and female gender (*P* = 0.002). In multivariable analysis, patients with visceral obesity were less likely to be male (OR = 0.070, *P* = 0.040) and showed less expression of CD68+PD-1+PD-L1+PD-L2+ (OR = 0.008, *P* = 0.010) in the normal liver. Individuals with sarcopenic obesity were more likely to have an elevated INR (International Ratio) (*P* = 0.038), had higher rate of LVI (*P* = 0.016) and a higher tumor grading (*P* = 0.049). In multivariate analysis, LVI (OR = 11.600, *P* = 0.014) was the single independent variable. More details indicating a notable association between immune cells and BC are displayed in Table 2. The detailed results of the analysis are shown in Supplementary Table 1. The major results are also summarized in Figure 1.

**Table 2:** Univariate Analysis and Multivariate Analysis of Body Composition With Multiplex Data in Intrahepatic Cholangiocarcinoma.

Outcome	Descriptive data		Univariate analysis		Multivariate analysis	
BMI	＜25 (n = 25）	≥25 (n = 23)	OR (95% CI)	*P*	OR (95% CI)	*P*
**LVI** (No/Yes (%); ref = No)	23 (92.0)/2 (8.0)	13 (56.5)/7 (30.4)	6.192 (1.117–34.316)	0.037	14.990 (1.657–135.594)	**0.016**
Tumor CD4 PD-1 (grouped by median; ref = low expression)	72.52 (7.82–376.98)	265.43 (125.53–1650.78)	4.148 (1.179–14.589)	0.027	2.933 (0.480–17.930)	0.244
Tumor CD4 PD-1 LAG3 TIM-3 (grouped by median; ref = low expression)	1.54 (0–22.362)	28.15 (4.27–285.49)	4.148 (1.179–14.589)	0.027	0.940 (0.030–29.849)	0.972
Tumor CD4 PD-1 TIM-3 (grouped by median; ref = low expression)	5.06 (0–99.83)	164.25 (28.34–815.10)	4.148 (1.179–14.589)	0.027	8.904 (1.615–49.088)	**0.012**
Tumor CD4 LAG3 TIM-3 (grouped by median; ref = low expression)	7.20 (1.41–55.62)	60.72 (22.19–334.85)	4.148 (1.179–14.589)	0.027	1.193 (0.154–9.217)	0.866
Tumor CD8 PD-1 TIM-3 (grouped by median; ref = low expression)	15.91 (2.59–61.59)	109.20 (25.80–1288.38)	4.148 (1.179–14.589)	0.027	2.418 (0.526–11.105)	0.256
Sarcopenia	No (n = 14)	Yes (n = 34)	OR (95% CI)	*P*	OR (95% CI)	*P*
**Sex** (male/female (%); ref = male)	10 (71.4)/4(28.6)	13 (38.2)/21 (61.8)	4.038 (1.047–15.581)	0.043	4.038 (1.047–15.581)	**0.043**
Myosteatosis	No (n = 20)	Yes (n = 28)	OR (95% CI)	*P*	OR (95% CI)	*P*
Sex (male/female (%); ref = male)	13 (65.0)/7 (35.0)	10 (35.7)/18 (64.3)	3.343 (1.006–11.107)	0.049	2.587 (0.437–15.312)	0.295
GGT U/L (≤100/>100; ref = ≤100)	188.00 (109.00–270.00)	83.00 (62.50–543.50)	0.178 (0.046–0.694)	0.013	0.182 (0.028–1.171)	0.073
Hemoglobin g/L (≤13/>13; ref = ≤13)	14.00 (12.20–14.80)	12.40 (12.00–14.20)	0.210 (0.058–0.760)	0.017	0.104 (0.016–0.691)	**0.019**
Tumor CD4 PD-1 LAG-3 (grouped by median; ref = low expression)	125.53 (15.207–951.33)	11.57 (0–40.58)	0.226 (0.062–0.825)	0.024	1.114 (0.121–10.234)	0.924
Tumor CD8 PD1-3 (grouped by median; ref = low expression)	837.17 (242.92–2066.75)	212.30 (425.66–518.91)	0.226 (0.062–0.825)	0.024	0.519 (0.072–3.748)	0.515
Tumor CD8 LAG-3 (grouped by median; ref = low expression)	1229.19 (406.82–3012.64)	336.65 (80.36–647.00)	0.226 (0.062–0.825)	0.024	0.289 (0.053–1.579)	0.152
Tumor CD8 TIM-3 (grouped by median; ref = low expression)	369.26 (223.21–2264.68)	125.41 (21.02–456.17)	0.143 (0.036–0.562)	0.005	0.118 (0.019–0.747)	**0.023**
VFA	＜100 (n = 22)	≥100 (n = 26)	OR (95% CI)	*P*	OR (95% CI)	*P*
**Sex** (male/female (%); ref = male)	5 (22.7)/17 (77.3)	18 (69.2)/8 (30.8)	0.131 (0.036–0.479)	0.002	0.070 (0.006–0.886)	**0.040**
Hemoglobin, g/L (≤13/>13; ref = ≤13)	12.30 (11.93–13.63)	14.05 (12.28–14.83)	5.250 (1.477–18.660)	0.010	11.011 (0.851–142.512)	0.066
Hospitalization days (≤14/>14; ref = ≤14)	16 (10–26)	12 (8–17)	0.303 (0.092–0.991)	0.048	3.438 (0.175–109.649)	0.368
Normal CD68 PD-1 (grouped by median; ref = low expression)	258.37 (61.11–5052.70)	32.72 (2.45–306.99)	0.182 (0.035–0.939)	0.042	0.348 (0.032–3.816)	0.388
Normal CD68 PD-1 PD-L1 PD-L2 (grouped by median; ref = low expression)	0 (0–39.76)	0 (0–0)	0.088 (0.009–0.893)	0.040	0.008 (0–0.323)	**0.010**
Sarcopenic obesity	No (n = 39)	Yes (n = 9)	OR (95% CI)	*P*	OR (95% CI)	*P*
INR (≤1/>1; ref = ≤1)	0.99 (0.94–1.03)	1.09 (1.03–1.13)	10.267 (1.143–92.256)	0.038	4.125 (0.374–45.520)	0.247
LVI (No/Yes (%); ref = No)	33 (84.6)/5 (12.8)	3 (33.3)/4 (44.4)	8.800 (1.502–51.556)	0.016	11.600 (1.659–81.102）	**0.014**
Tumor grading ((G1/G2)/(G3/G4) (%); ref = G1/G2)	31 (79.5)/6 (15.4)	4 (44.4)/4 (44.4)	5.167 (1.004–26.597)	0.049	3.107 (0.372–25.953)	0.295

Data presented as median and interquartile range if not noted otherwise. The multiplex data was divided into high and low expression groups based on the median. The table only shows the data with *P* value < 0.05 in the univariate analysis. Variables displaying a *P* value < 0.05 in the univariate analysis were transferred into a multivariable logistic regression model.

BMI, body mass index; GGT, gamma-glutamyl transferase; INR, international normalized ratio; LVI, lymph vascular invasion; OR, odds ratio; VFA, visceral fat area.

Multiplex date (×10^−5^).

### Association of Body Composition With Immune Cells in Perihilar Cholangiocarcinoma

Within the pCCA group, 22 (45.8%) patients were classified as obese, 27 (54.0%) showed sarcopenia, 7 (14.6%) displayed sarcopenic obesity, 23 (47.9%) showed myosteatosis and 27 (56.3%) displayed visceral obesity (Table 1). Similar to iCCA, the association between BC characteristics and clinical characteristics as well as immune cell composition, univariate und multivariable logistic regressions were conducted.

**Figure 2:**
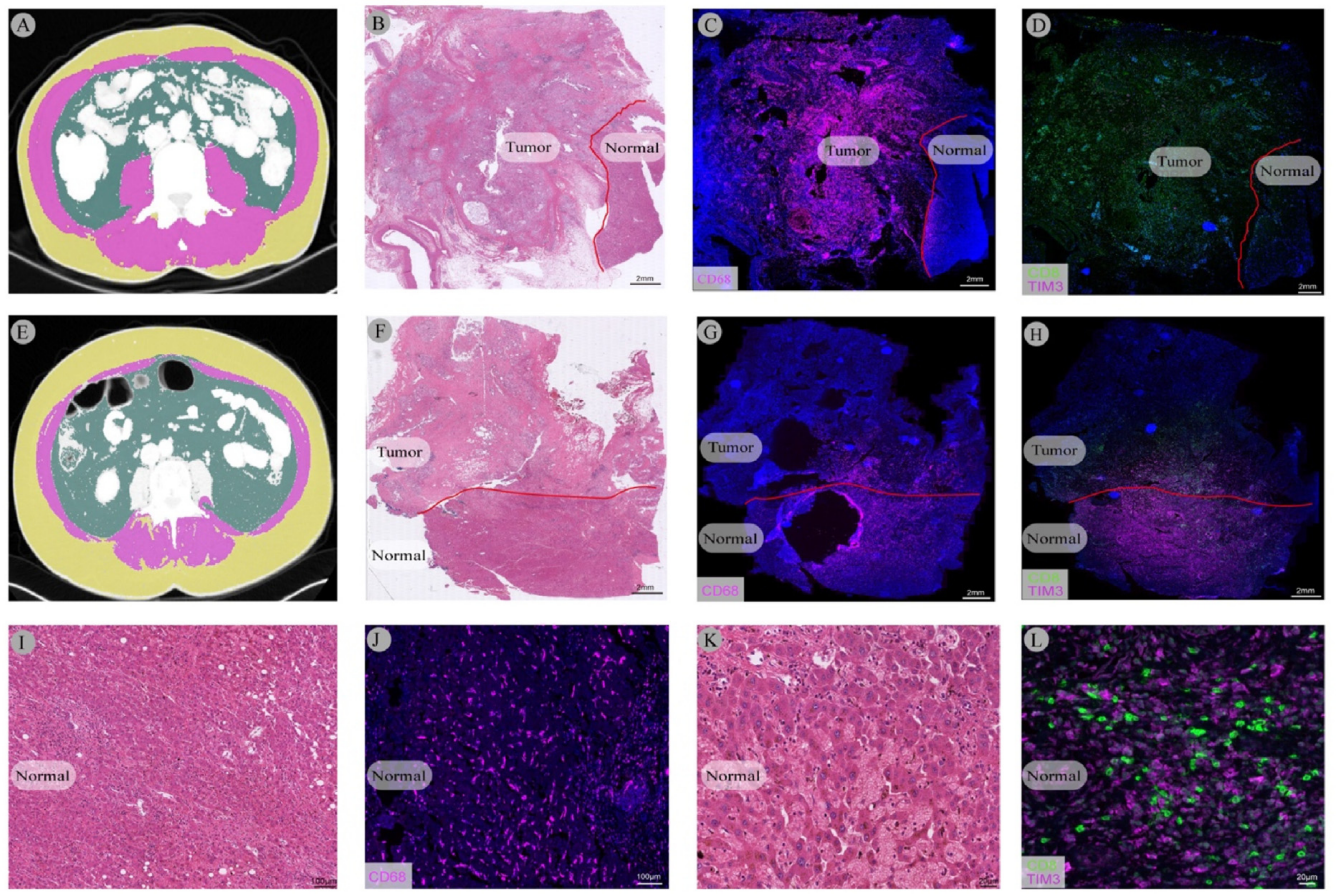
**Immune cell composition in perihilar cholangiocarcinoma with respect to sarcopenia**. Immune cells composition is demonstrated in two patients with perihilar cholangiocarcinoma. (A) CT-scan displaying regular muscle mass and no signs of sarcopenia. (B) Whole-slide H&E staining with tumor and normal liver compartment of the patient. (C, D) Whole-slide multiplex imaging displaying low expression of CD68+ cells and CD8+TIM3+ cells in the normal liver. (E) CT-scan displaying sarcopenia. (F) Whole-slide H&E staining with tumor and normal liver compartment of the patient. (G, H) Whole-slide multiplex imaging displaying increased expression of CD68+ cells and CD8+TIM3+ cells in the normal liver. (I, J, K, L)) Zoomed-in images (H&E, multiplex) slide of the normal liver displaying increased expression of CD68+ cells and CD8+TIM3+ cells in patient E. H&E, hematoxylin and eosin.

**Figure 3:**
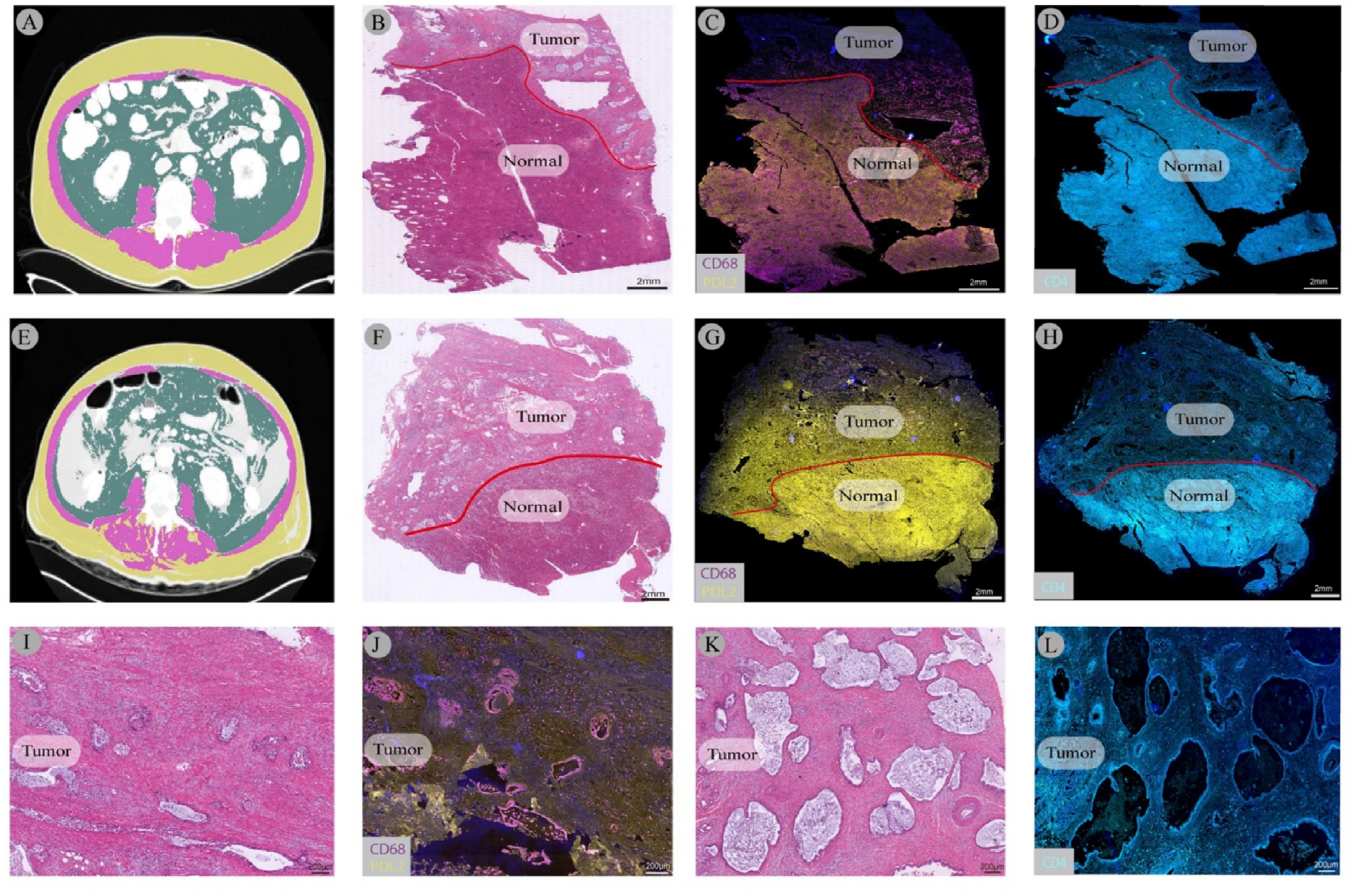
**Immune cell composition in perihilar cholangiocarcinoma with respect to myosteatosis**. Immune cells composition is demonstrated in two patients with perihilar cholangiocarcinoma. (A) CT-scan displaying no signs of myosteatosis. (B) Whole-slide H&E staining with tumor and normal liver compartment of the patient. (C, D) Whole-slide multiplex imaging displaying of low expression of CD68+PD-L2+cells and high expression of CD4+ cells in tumor tissues. (E) CT-scan displaying myosteatosis. (F) Whole-slide H&E staining with tumor and normal liver compartment of the patient. (G, H) Whole-slide multiplex imaging displaying of high expression of CD68+PD-L2+cells and low expression of CD4+ cells in tumor tissues. (I, J, K, L) Zoomed-in images (H&E, multiplex) slide of the tumor tissues displaying increased high expression of CD68+PD-L2+cells and low expression of CD4+ cells in patient E. H&E, hematoxylin and eosin.

Here, patients with obesity were less likely to have lymph node metastases (OR = 0.254, *P* = 0.026) and did not show association with immune cell composition. In contrast, the individuals with sarcopenia were more likely to be female (*P* = 0.001) and showed higher expression of CD68+ (*P* = 0.038) and CD8+TIM-3+ (*P* = 0.023) cells in the normal liver. In multivariable analysis, patients with sarcopenia showed higher CD68+ (OR = 13.581, *P* = 0.018) and CD8+TIM-3+ (OR = 20.324, *P* = 0.021) expression in the normal liver. PCCA patients with myosteatosis displayed higher expression of CD68+PD-L2+ (*P* = 0.024) and reduced CD4+ (*P* = 0.040) expression in tumor tissue. In multivariable analysis, patients with myosteatosis showed higher CD68+PD-L2+ (OR = 8.509, *P* = 0.013) and lower CD4+ (OR = 0.118, *P* = 0.013) expression in tumor tissue. Individuals with visceral obesity displayed reduced CD4+ICOS+TIGIT+CTLA-4+ (*P* = 0.041), CD4+TIGIT+ (*P* = 0.041), CD8+ICOS+TIGIT+CTLA-4+ (*P* = 0.041), CD8+TIGIT+ (*P* = 0.041), CD8+ICOS+CTLA-4+ (*P* = 0.041), CD8+TIGIT+CTLA-4+(*P* = 0.041) cells in tumor tissue and lower expression of CD4+ICOS+TIGIT+ (*P* = 0.041) cells in the normal liver. In the multivariable analysis, lower CD4+ICOS+TIGIT+ (OR = 0.179, *P* = 0.032) expression in the normal liver was the single independent variable. Individuals with sarcopenic obesity displayed higher expression of CD8+PD1+PDL1+PDL2+ (*P* = 0.026) in the normal liver as well as shorter hospitalization (*P* = 0.047). In multivariable analysis, higher CD8+PD1+PDL1+PDL2+ (OR = 15.884, *P* = 0.024) expression was the single independent variable. More details are displayed in Table 3. The detailed results are shown in Supplementary Table 2. The major results are also summarized in Figure 1. Exemplary multiplexed images and corresponding BC for patients with sarcopenia and myosteatosis are presented in Figure 2, Figure 3.

**Table 3:** Univariate Analysis and Multivariate Analysis of Body Composition With Multiplex Data in Perihilar Cholangiocarcinoma.

Outcome	Descriptive data		Univariate analysis		Multivariate analysis	
BMI	＜25 (n = 26）	≥25 (n = 22)	OR (95% CI)	*P*	OR (95% CI)	*P*
**pN category** (N0/N1 (%); ref = N0)	8 (30.8)/18 (69.2)	14 (63.6)/8 (36.4)	0.254 (0.076–0.846)	0.026	0.254 (0.076–0.846)	**0.026**
Sarcopenia	No (n = 21)	Yes (n = 27)	OR (95% CI)	*P*	OR (95% CI)	*P*
Sex (male/female (%); ref = male)	19 (90.5)/2 (9.5)	10 (37.0)/17 (63.0)	16.150 (3.092–84.361)	0.001	5.523 (0.410–62.548)	0.190
Normal CD68 (grouped by median, ref = low expression)	7183.65 (2857.17–23921.77)	16799.60 (7070.04–26386.84)	5.25 (1.093–7.437)	0.038	13.581 (1.572–117.294)	**0.018**
Normal CD8 TIM-3 (grouped by median, ref = low expression)	84.25 (0–454.03)	1.43 (0–881.64)	13.333 (1.434–123.989）	0.023	20.324 (1.579–261.608)	**0.021**
Myosteatosis	No (n = 25)	Yes (n = 23)	OR (95% CI)	*P*	OR (95% CI)	*P*
Tumor CD68 PD-L2 (grouped by median, ref = low expression)	527.67 (4.27–1281.34)	925.33 (23.90–1902.77)	4.375 (1.210–15.812)	0.024	8.509 (1.570–46.118)	**0.013**
Tumor CD4 (grouped by median, ref = low expression)	3059.79 (762.03–3749.18)	2481.31 (359.70–4439.26)	0.215 (0.058–0.806)	0.023	0.118 (0.022–0.637)	**0.013**
VFA	≤100 (n = 21)	＞100 (n = 27)	OR (95% CI)	*P*	OR (95% CI)	*P*
Tumor CD4 ICOS TIGIT CTLA (grouped by median, ref = low expression)	0 (0–20.32)	8.27 (0–28.75)	0.250 (0.066–0.946)	0.041	8.530 (0.241–302.501)	0.239
Tumor CD4 TIGIT (grouped by median, ref = low expression)	58.25 (2.80–204.79)	46.30 (5.89–126.62)	0.250 (0.066–0.946)	0.041	0.509 (0.083–3.130)	0.466
Tumor CD8 ICOS TIGIT CTLA (grouped by median, ref = low expression)	7.88 (0–128.20)	7.44 (0–49.75)	0.25 (0.066–0.946)	0.041	n.a.	1
Tumor CD8 TIGIT (grouped by median, ref = low expression)	153.84 (38.09–573.73)	153.79 (12.29–333.11)	0.25 (0.066–0.946)	0.041	1.562 (0.053–46.293)	0.797
Tumor CD8 ICOS CTLA (grouped by median, ref = low expression)	14.26 (0–960.67)	9.10 (0–157.62)	0.25 (0.066–0.946)	0.041	4.977 (0.167–148.397)	0.354
Tumor CD8 TIGIT CTLA (grouped by median, ref = low expression)	18.29 (0–40.595)	17.38 (1.27–150.57)	0.25 (0.066–0.946)	0.041	0.276 (0.048–1.584)	0.149
Normal CD4 ICOS TIGIT (grouped by median, ref = low expression)	0 (0–80.02)	0(0–0)	0.179 (0.037–0.863）	0.032	0.153 (0.028–0.833)	**0.030**
Sarcopenic obesity	No (n = 41)	Yes (n = 7)	OR (95% CI)	*P*	OR (95% CI)	*P*
Hospitalization, days (≤14/>14(%); ref = ≤14)	15 (9–24)	13 (10–20)	0.182 (0.034–0.982)	0.047	0.153 (0.014–1.732)	0.130
Normal CD8 PD1 PD-L1 PD-L2 (grouped by median, ref = low expression)	0 (0–0)	0 (0–61.17)	9.200 (1.304–64.895）	0.026	15.884 (1.447–174.360）	**0.024**

Data presented as median and interquartile range if not noted otherwise. The multiplex data was divided into high and low expression groups based on the median. The table only shows the data with *P* value < 0.05 in the univariate analysis. Variables displaying a *P* value < 0.05 in the univariate analysis were transferred into a multivariable logistic regression model.

BMI, body mass index; GGT, gamma-glutamyl transferase; INR, international normalized ratio; LVI, lymph vascular invasion; OR, odds ratio.

Note: multiplex date (×10−^5^).

### Association of Cancer-specific and Recurrence-free Survival With Body Composition, Immune Cell Infiltration and Clinico–pathological Variables in Intrahepatic Cholangiocarcinoma

To investigate the association between oncological outcome, univariate and multivariable Cox regressions were conducted in including the parameters of BC, the immune cell infiltrates which were associated to BC in the preceding univariate analysis (CD4+PD-1+, CD4+PD-1+LAG3+, CD4+PD-1+TIM-3+, CD4+LAG3+TIM-3+, CD8+ PD-1+TIM-3+, CD4+,CD4+PD1+LAG3+, CD8+PD1+, CD8+LAG3+, CD8+TIM-3+, CD68+PD-1+, CD68+ PD-1+PD-L1+PD-L2+), and the clinicopathological data. For RFS, intraoperative packed red blood cells (PRBC) (*P* = 0.044), lymph node metastases (*P* = 0.004), myosteatosis (*P* = 0.017) were associated in univariate analysis. All variables showing *P* value < 0.05 were included in a multivariable Cox regression model. Here, PRBC (hazard ratio [HR] = 2.683, *P* = 0.033) and LVI (HR = 2.579, *P* = 0.019) were the independent prognostic variables for RFS. For CCS, intraoperative fresh frozen plasma (FFP) (*P* = 0.030), lymph node metastases (*P* = 0.001), intensive care unit (ICU) days (*P* = 0.009), visceral obesity (*P* = 0.032), CD4+PD-1+cells in tumor tissue (*P* = 0.035) showed a significant association in univariate analysis. In the corresponding multivariable model, intraoperative FFP (HR = 2.519, *P* = 0.020), lymph node metastases (HR = 3.688, *P* = 0.003), CD4+PD-1+cells in tumor tissue (HR = 2.733, *P* = 0.011) were the independent prognostic variables for CCS. More details are provided in Table 4.

**Table 4:** Analysis of Recurrence-free Survival and Cancer-specific Survival in Intrahepatic Cholangiocarcinoma.

Variables	Recurrence-free survival				Cancer-specific survival			
	Univariable		Multivariable		Univariable		Multivariable	
	HR（95% CI）	*P*	HR（95% CI）	*P*	HR（95% CI）	*P*	HR（95% CI）	*P*
Sex (male = 1)	0.861 (0.441–1.679)	0.660			0.642 (0.323–1.277)	0.207		
Age, years (≤65 = 1)	0.833 (0.428–1.619)	0.589			1.281 (0.640–2.563)	0.485		
PVE (no = 1)	1.087 (0.330–3.580)	0.892			0.776 (0.186–3.247)	0.729		
ASA (I/II = 1)	0.708 (0.362–1.385）	0.313			1.081 (0.546–2.143）	0.822		
Neoadjuvant therapy (no = 1)	2.339 (0.707–7.740)	0.164			1.525 (0.362–6.418)	0.565		
AST, U/l (≤40 = 1)	0.998 (0.513–1.941)	0.995			1.292 (0.652–2.559)	0.463		
ALT, U/l (≤40 = 1)	1.369 (0.625–2.997)	0.432			0.788 (0.307–2.019)	0.619		
GGT, U/l (≤100 = 1)	1.757 (0.850–3.630)	0.128			1.480 (0.708–3.092)	0.297		
Bilirubin, mg/dl (≤1 = 1)	0.903 (0.391–2.084)	0.811			1.130 (0.507–2.517)	0.765		
Platelet count, L/nl (≤250 = 1)	0.821 (0.414–1.626)	0.571			0.738 (0.368–1.480)	0.392		
Prothrombin time (≤110 = 1)	0.449 (0.172–1.172）	0.102			0.436 (0.152–1.256)	0.124		
INR (≤1 = 1)	1.409 (0.701–2.832)	0.355			1.407 (0.694–2.855)	0.344		
Hemoglobin, g/dl (≤13 = 1)	0.782 (0.395–1.550)	0.482			0.732 (0.365–1.466)	0.378		
CRP, mg/l (≤10 = 1)	1.825 (0.892–3.733)	0.099			1.755 (0.851–3.619)	0.127		
Operative time, min (≤360 = 1)	1.568 (0.673–3.654)	0.297			2.163 (0.971–4.819)	0.059		
Intraoperative PRBC (No = 1)	**1.992 (1.019–3.895)**	**0.044**	**2.683 (1.085–6.633)**	**0.033**	1.784 (0.900–3.537)	0.097		
Intraoperative FFP (no = 1)	1.742 (0.888–3.417)	0.106			**2.154 (1.075–4.314)**	**0.030**	2.519 (1.154–2.498)	**0.020**
R1 resection (R0 = 1)	1.109 (0.336–3.657)	0.865			1.086 (0.331–3.564)	0.891		
MVI (no = 1)	1.663 (0.852–3.246)	0.136			1.121 (0.561–2.240)	0.747		
LVI (no = 1)	2.218 (0.987–4.984)	0.054			2.017 (0.850–4.782)	0.111		
Tumor grading (G1/G2 = 1)	2.153 (1.976–4.749)	0.057			2.135 (0.971–4.698)	0.059		
pN category (N0 = 1)	**3.110 (1.436–6.733)**	**0.004**	2.579 (1.166–5.706)	0.019	**3.384 (1.622–7.074)**	**0.001**	**3.688 (1.539–8.837)**	**0.003**
ICU time, days (≤1 = 1)	1.480 (0.734–2.983)	0.273			**2.572 (1.266**–**5.222**）	**0.009**	1.479 (0.568–3.850)	0.423
Hospitalization, days (≤14 = 1)	1.717 (0.880–3.353)	0.113			1.354 (0.683–2.683)	0.386		
Perioperative complications (Clavien–Dindo 0/I/II = 1)	1.634 (0.826–3.232)	0.158			1.417 (0.704–2.850)	0.329		
Adjuvant therapy (No = 1)	1.823 (0.881–3.772）	0.105			1.514 (0.716–3.204）	0.278		
BMI, kg/m2 (≤25 = 1)	1.034 (0.943–1.132)	0.479			1.585 (0.796–3.159)	0.081		
Visceral_fat area (≤100 = 1)	1.128 (0.579–2.198)	0.723			**2.182 (1.067–4.461)**	**0.032**	2.132 (0.900–5.053)	0.085
Sarcopenia（no = 1）	0.804 (0.393–1.642)	0.549			1.005 (0.477–2.120)	0.990		
Myosteatosis（no = 1）	**0.431 (0.217–0.860)**	**0.017**	0.448 (0.198–1.017)	0.055	0.691 (0.348–1.372)	0.291		
Sarcopenic obesity（no = 1）	1.263 (0.550–2.902)	0.583			1.329 (0.545–3.237)	0.532		
Tumor CD4 PD-1^a^	1.214 (0.611–2.412)	0.579			**2.183 (1.057–4.508)**	**0.035**	**2.733 (1.256–5.948)**	**0.011**
Tumor CD4 PD-1 LAG-3 TIM-3^a^	0.996 (0.502–1.977）	0.990			1.367 (0.668–2.797)	0.392		
Tumor CD4 PD-1 TIM-3^a^	0.894 (0.451–1.774)	0.749			1.431 (0.700–2.926)	0.326		
Tumor CD4 LAG3 TIM-3^a^	1.400 (0.689–2.808)	0.343			2.085 (0.993–4.378)	0.052		
Tumor CD8 PD-1 TIM-3^a^	1.740 (0.872–3.475)	0.116			1.638 (0.800–3.352)	0.177		
Tumor CD4 PD-1 LAG-3^a^	1.494 (0.746–2.990)	0.257			1.721 (0.834–3.551)	0.142		
Tumor CD8 PD-1^a^	1.685 (0.840–3.380)	0.142			1.485 (0.725–3.045)	0.280		
Tumor CD8 LAG-3^a^	1.452 (0.725–2.905)	0.292			1.852 (0.891–3.852)	0.099		
Tumor CD8 TIM-3^a^	2.002 (0.986–4.062)	0.055			1.441 (0.703–2.955)	0.319		
Normal CD68 PD-1^a^	0.667 (0.275–1.619)	0.371			0.508 (0.207–1.243)	0.138		
Normal CD68 PD-1 PD-L1 PD-L2^a^	0.513 (0.150–1.756)	0.288			0.531 (0.155–1.827)	0.316		

Variables displaying a *P* value < 0.05 in the univariate Cox Regression were transferred into a multivariable Cox regression model. Bold values indicate statistical significance.

ALT, alanine aminotransferase; ASA, American Society of Anesthesiologists; AST, aspartate aminotransferase; BMI, body mass index; CRP, C-reactive protein; FFP, fresh frozen plasma; GGT, gamma-glutamyl transferase; HR, hazard ratio; iCCA, intrahepatic cholangiocarcinoma; ICU, intensive care unit; INR, international normalized ratio; LVI, lymph vascular invasion; MVI, microvascular invasion; PRBC, packed red Blood Cells; PVE, portal vein embolization; RFS, recurrence-free survival.

a All multiplex data are grouped by median expression and referenced to low expression.

### Association of Cancer-specific and Recurrence-free Survival With Body Composition, Immune Cell Infiltration and Clinico–pathological Variables in Perihilar Cholangiocarcinoma

To investigate the association between oncological outcome, univariate, and multivariable Cox regressions were conducted in including the parameters of BC, the immune cell infiltrates which were associated with BC in the preceding univariate analysis (CD68+, CD8 TIM3+, CD68 PDL2+, CD4+, CD4+ICOS+TIGIT+CTLA-4+, CD4+TIGIT+, CD8+ICOS+TIGIT+CTLA-4+, CD8+TIGIT+, CD8+ICOS+CTLA-4+, CD8+TIGIT+CTLA-4+, CD4+ICOS+TIGIT+, CD8+PD-1+PD-L1+PD-L2+), and the clinicopathological data set. For RFS, R1 resection (*P* = 0.009), intraoperative PRBC (*P* = 0.047), intraoperative FFP (*P* = 0.007), tumor grading (*P* = 0.008), ICU days (*P* = 0.011), and CD8+TIGIT+cells (*P* = 0.044) in tumor tissue were associated in univariate analysis and all variables showing *P* value < 0.05 were included in a multivariable Cox regression model. Here, intraoperative FFP (HR = 4.262, *P* = 0.010) and tumor grading (HR = 2.790, *P* = 0.036) were the independent prognostic variables for RFS. For CCS, intraoperative FFP (*P* = 0.011), R1 resection (*P* = 0.035), tumor grading (*P* = 0.022), ICU days (*P* = 0.009), and CD8+TIGIT+cells (*P* = 0.040) in tumor tissue showed a significant association in univariate analysis. In the corresponding multivariable model, intraoperative FFP (HR = 2.881, *P* = 0.024) were the independent prognostic variables for CCS (Table 5).

**Table 5:** Analysis of Recurrence-free Survival and Cancer-specific Survival in Perihilar Cholangiocarcinoma.

Variables	Recurrence-free survival				Cancer-specific survival			
	Univariable		Multivariable		Univariable		Multivariable	
	HR (95%)	*P*	HR (95%)	*P*	HR（95%)	*P*	HR (95%)	*P*
Sex (Male = 1)	1.295 (0.640–2.620)	0.472			1.287 (0.656–2.525)	0.463		
Age, years (≤65 = 1)	0.781 (0.389–1.566)	0.486			1.078 (0.552–2.103)	0.826		
PVE (no = 1)	0.891 (0.443–1.795)	0.748			0.890 (0.454–1.744)	0.735		
ASA (I/II = 1)	1.283 (0.635–2.591）	0.487			1.298 (0.661–2.550）	0.448		
Neoadjuvant therapy (no = 1)	0.672 (0.160–2.817)	0.587			0.336 (0.046–2.457)	0.283		
AST, U/l (≤40 = 1)	0.896 (0.436–1.843)	0.765			0.974 (0.488–1.944)	0.941		
ALT, U/l (≤40 = 1)	0.844 (0.411–1.733)	0.643			1.262 (0.591–2.698)	0.547		
GGT, U/l (≤100 = 1)	0.751 (0.225–2.502)	0.641			0.814 (0.246–2.689)	0.736		
Bilirubin, mg/dl (≤1 = 1)	1.013 (0.502–2.044)	0.972			1.275 (0.656–2.481)	0.474		
Platelet count, 1/nl (≤250 = 1)	1.017 (0.437–2.364)	0.969			0.732 (0.349–1.537)	0.410		
Prothrombin time (≤110)	0.536 (0.219–1.308）	0.171			0.532 (0.220–1.287)	0.162		
INR (≤1 = 1)	1.634 (0.783–3.408)	0.190			1.719 (0.839–3.521)	0.139		
Hemoglobin, g/dl (≤13 = 1)	0.584 (0.252–1.356)	0.211			0.718 (0.336–1.534)	0.392		
CRP, mg/l (≤10 = 1)	0.918 (0.448–1.881)	0.815			1.213 (0.610–2.415)	0.582		
Operative time, min (≤360 = 1)	1.536 (0.725–3.256)	0.262			1.461 (0.712–2.997)	0.301		
Intraoperative PRBC (no = 1)	**2.088 (1.011–4.312)**	**0.047**	0.391 (0.131–1.173)	0.094	1.992 (0.994–3.991)	0.052		
Intraoperative FFP (no = 1)	**3.344 (1.406–8.452)**	**0.007**	**4.262 (1.423–12.763)**	**0.010**	**2.980 (1.291–6.882)**	**0.011**	2.881 (01.148–7.230)	**0.024**
R1 resection (R0 = 1)	**3.027 (1.321–6.935)**	**0.009**	1.226 (0.350–4.296)	0.750	**2.394 (1.065–5.383)**	**0.035**	1.194 (0.445–3.201)	0.725
MVI (no = 1)	2.131 (0.997–4.553)	0.051			1.935 (0.935–4.006）	0.075		
LVI (no = 1)	2.051 (0.955–4.406)	0.066			1.568 (0.721–3.407)	0.256		
Tumor grading (G1/G2 = 1)	**3.098 (1.345–7.135)**	**0.008**	**2.790 (1.069–7.284)**	**0.036**	**2.601 (1.145–5.909)**	**0.022**	1.789 (0.696–4.602)	**0.228**
pN category (N0 = 1)	1.887 (0.926–3.846)	0.080			1.736 (0.879–3.429)	0.112		
ICU time, days (≤1 = 1)	**2.612 (1.245–5.479)**	**0.011**	1.976 (0.668–5.846)	0.219	2.572 (1.266–5.222)	**0.009**	1.329 (0.542–3.262)	0.534
Hospitalization, days (≤14 = 1)	1.177 (0.528–2.623)	0.690			1.405 (0.613–3.222)	0.422		
Perioperative complications (Clavien–Dindo 0/I/II = 1)	1.476 (0.717–3.037)	0.290			1.716 (0.852–3.457)	0.131		
Adjuvant therapy (No = 1)	2.062 (0.965–4.405）	0.062			1.391 (0.645–3.001)	0.400		
BMI, kg/m2 (≤25 = 1)	0.989 (0.922–1.062)	0.763			1.372 (0.703–2.680)	0.354		
Visceral_fat area (≤100 = 1)	1.161 (0.571–2.360)	0.679			1.160 (0.593–2.270)	0.665		
Sarcopenia（no = 1）	1.762 (0.860–3.611)	0.122			1.979 (0.982–3.986)	0.056		
Myosteatosis（no = 1）	1.160 (0.578–2.327)	0.676			1.346 (0.691–2.622)	0.382		
Sarcopenic obesity（no = 1）	1.353 (0.519–3.526）	0.537			1.178 (0.453–3.058）	0.737		
Normal CD68^a^	0.913 (0.377–2.210)	0.840			1.406 (0.615–3.217)	0.420		
Normal CD8 TIM-3^a^	0.945 (0.395–2.261)	0.899			0.828 (0.359–1.908)	0.658		
Tumor CD68 PD-L2^a^	0.857 (0.401–1.830)	0.689			0.784 (0.382–1.610)	0.507		
Tumor CD4^a^	0.839 (0.382–1.842)	0.662			0.759 (0.366–1.575)	0.459		
Tumor CD4 ICOS TIGIT CTLA-4^a^	0.553 (0.247–1.237)	0.149			0.577 (0.275–1.210)	0.146		
Tumor CD4 TIGIT^a^	1.104 (0.503–2.422)	0.806			1.020 (0.491–2.121)	0.957		
Tumor CD8 ICOS TIGIT CTLA-4^a^	0.624 (0.282 = 1.379)	0.244			0.635 (0.305–1.324)	0.226		
Tumor CD8 TIGIT^a^	**0.431 (0.190–0.977)**	**0.044**	0.526 (0.211–1.313)	0.169	**0.454 (0.214–0.963)**	**0.040**	0.464 (0.207–1.041)	0.062
Tumor CD8 ICOS CTLA-4^a^	0.798 (0.363–1.753)	0.575			0.778 (0.375–1.617)	0.502		
Tumor CD8 TIGIT CTLA-4^a^	0.462 (0.967–4.855)	0.060			0.507 (0.242–1.064)	0.072		
Normal CD4 ICOS TIGIT^a^	1.053 (0.394–2.815)	0.918			1.409 (0.598–3.318)	0.433		
Normal CD8 PD-1 PD-L1 PD-L2^a^	0.298 (0.069–1.291)	0.106			0.692 (0.256–1.870)	0.468		

Variables displaying a *P* value < 0.05 in the univariate Cox Regression were transferred into a multivariable Cox regression model. Bold values indicate statistical significance.

ALT, alanine aminotransferase; ASA, American Society of Anesthesiologists; AST, aspartate aminotransferase; BMI, body mass index; CRP, C-reactive protein; FFP, fresh frozen plasma; GGT, gamma-glutamyl transferase; HR, hazard ratio; iCCA, intrahepatic cholangiocarcinoma; ICU, intensive care unit; INR, international normalized ratio; LVI, lymph vascular invasion; MVI, microvascular invasion; PRBC, packed red Blood Cells; PVE, portal vein embolization; RFS, recurrence-free survival.

a All multiplex data are grouped by median expression and referenced to low expression.

### Subgroup Analysis for CD8+ T Cell Density

We performed a subgroup analysis comparing clinical and pathological features between patients with high and low CD8+ T cell density for both iCCA and pCCA. In iCCA patients, significant differences were observed between the high and low CD8+ T cell density groups in terms of prothrombin time (*P* = 0.042) and international normalized ratio (INR, *P* = 0.036) However, no statistically significant differences were found between the two groups in other clinical parameters, including length of hospital stay and postoperative recurrence rates (Supplementary Table 3). In pCCA patients, all clinical and pathological variables did not differ significantly between the two groups (Supplementary Table 4).

## DISCUSSION

CCA is a rare but highly invasive malignancy. In recent years, immunotherapy has been proven to show some benefits in the treatment for CCA. However, it is not effective for all patients with advanced CCA.^12^ Therefore, further research is needed to understand which patients are suitable for immunotherapy and how to improve this treatment method to enhance its effectiveness and efficiency. Given the frequent changes in BC in patients with CCA, we here systematically investigated the association between BC parameters and immune cell characteristics within the tumor and normal liver tissue using whole-slide computer-based multiplexed imaging. To the best of our knowledge, this is the first structural investigation indicating a meaningful association between immune cells and BC in CCA.

### Key Findings

Our analysis identified significant alterations in BC among CCA patients. Specifically, the prevalence of obesity, sarcopenia, and visceral obesity was notable across both cancer subtypes. Importantly, these alterations in BC were not merely epidemiological but were intricately linked with distinct immune cell populations.

In the iCCA group, myosteatosis demonstrated a marked association with TIM-3+CD8+ cells within the tumor compartment, highlighting a potential immune suppression mechanism at play in this patient subgroup. Furthermore, obesity in iCCA was correlated with an increased expression of PD-1+TIM-3+CD4+ cells, suggesting an interaction between BC and immune checkpoint markers, which might have an influence on immunotherapy responses.

In pCCA, a similar pattern emerged, with myosteatosis being linked to PD-L2+CD68-macrophages and CD4+ cells in the tumor, while sarcopenia was associated with CD68+ cells and TIM-3+CD8+ cells in the liver parenchyma. These findings suggest that BC alterations in CCA patients are not only prevalent but may serve as biomarkers for immune cell infiltration and function, potentially affecting tumor behavior and response to therapy.

Moreover, immune cell infiltration patterns differed between the tumor microenvironment and the normal liver tissue, underlining the complex role BC plays in modulating both local and systemic immune responses in CCA. These insights provide a novel understanding of how BC might influence immune cell dynamics and suggest further research into targeted therapies based on the patient's BC profile.

### Body Composition is Frequently Altered in Patients With Perihilar and Intrahepatic Cholangiocarcinoma

Altered BC is commonly observed in individuals suffering from cancer, especially in western patients which already have a high prevalence of overweight patients and obesity.^13^ Historically, BMI has been the measurement of interest in cancer patients as it is easily assessable and showed the so-called obesity paradox meaning obese patients were more prone to develop malignancies but showed better overall survival than normal weight patients. This paradox is nowadays explained by the correlation with higher residuals muscle mass in obese patients and more sophisticated measures of BC e.g. sarcopenia, myosteatosis, sarcopenic obesity or visceral fat/obesity have been developed to describe BC in cancer patients. As such, Prado *et al.* demonstrated sarcopenia as an independent risk factor in individuals with obesity with gastrointestinal and pulmonary malignancies.^14^ Given the clinical implications of altered BC as impaired oncological outcome and also increased perioperative complications, international guidelines already recommend an assessment of BC in chronic liver disease.

While BC, especially sarcopenia, has been investigated in HCC, only a few reports in the literature describe the clinical value in CCA.^15^ In a heterogenous data set of 117 patients with biliary tract cancer, sarcopenia, and myosteatosis were independently prognostic for survival.^16^ Another study of 75 palliative CCA also indicated a prognostic role of both sarcopenia and myosteatosis^17^ showing the clinical importance of BC in CCA. However, a large cohort did not display an association between BC and oncological outcome in patients undergoing curative-intent surgery but underlined the prevalence of altered BC.^18^ As described above, all measures of BC were frequently altered in our study cohort demonstrates the prevalence of this phenomenon in CCA and is also in line with the literature.^16^^,^^17^

### Body Composition is Associated With Immune Cell Infiltration Within the Tumor and Normal Liver Parenchyma

Of note, our data reveal a notable association between BC and immune cells in the tumor and normal liver parenchyma and showed statistical significance in multivariable models including a large set of clinical (preoperative, intraoperative and postoperative) and pathological characteristics. This illustrates a strong association between BC and immune cells. Interestingly, the most relevant association regarding tumor-infiltrating immune cells and BC was observed for myosteatosis being significantly associated with reduced TIM-3+CD8+cells in iCCA and reduced CD4+ and a higher expression of PDL2+CD68+cells in pCCA. In contrast, sarcopenia, sarcopenic obesity and visceral obesity showed more associations with distinct immune cell subsets in the normal liver parenchyma.

There is a growing body of research implying the liver to take part in the realm of cancer cachexia. During cancer progression, hepatocytes not only increase the synthetization of acute-phase proteins which drives muscle protein degradation but also downregulate the excretion of interleukins (ILs). For example, IL-4, an anti-inflammatory, anti-cachectic cytokine, is downregulated in the liver of patients of pancreatic cancer.^19^ Moreover, in patients with pancreatic cancer and cachexia, the infiltration of macrophages into liver tissue has been shown to trigger liver parenchymal cells to induce the production of proinflammatory cytokines resembling IL-6.^20^ Also, a positive correlation between levels of CD68 mRNA and secretion of TNF—another proinflammatory cytokine associated with cancer cachexia—has been demonstrated in *in vitro* experiments.^21^

Of note, our study demonstrated subtypes of CD68+ macrophages in the normal liver parenchyma to be expressed in patients with sarcopenia in pCCA as well as in individuals with visceral obesity in iCCA. In adipose tissues of cachexia patients, activated CD8+ T cells have already been identified as specific contributors to the upregulation of interferon-γ (IFNγ) expression, exhibiting a considerable pro-catabolic impact on adipocytes *in vitro*.^22^ Also, abundance of CD8+ T cells inversely correlates with elements of muscle catabolism.^23^ In line with these observations in other settings, we also observed a higher expression of CD8+TIM-3+ T cells in the normal tissues of pCCA patients diagnosed with sarcopenia compared with non-sarcopenic patients.

### Body Composition-associated Immune Cell Infiltration in Relation to Oncological Prognosis

As described above, myosteatosis was associated with reduced TIM-3+CD8+cells in iCCA and CD4+ and PDL2+CD68+cells in pCCA within the tumor tissue itself. Further, myosteatosis was recently linked to 75% greater mortality risk in various cancers which was specifically based on worse overall survival in patients with gynecological, renal, periampullary/pancreatic, hepatocellular, gastroesophageal, colorectal carcinoma, and lymphomas.^24^ Interestingly, the observed immune cell infiltrates in patients with myosteatosis have been associated to oncological outcome before.

T cell immunoglobulin and mucin domain-3 (TIM-3) is an inhibitory immune checkpoint protein widely expressed on CD4+ and CD8+cells of the innate immune system.^25^ Combining TIM-3 with its ligand results in T cell and NK cell exhaustion and is a negative immune function regulator.^25^ When TIM-3 protein is highly expressed on the surface of CD8+ T cells, CD8+ T cell function is suppressed and production of the inflammatory factor IFN-γ is reduced.^26^ Numerous publications have demonstrated that TIM-3 overexpression was correlated with increased aggressive disease and poor survival in solid tumors.^27^ This phenomenon may have been attributable to the exhaustion of T cell function via the TIM-3/Gal-9 pathway. Interestingly, patients with myosteatosis showed a lower expression of TIM-3+CD8+cells in iCCA tumors which might explain the mixed results of the prognostic influence of myosteatosis in the literature.^16^, ^17^, ^18^ In contrast, TIM-3+CD4+PD1+cells within the tumor tissue were enriched in obese patients defined by BMI.

While recent studies show programmed death ligand-2 (PD-L2), another ligand of PD-1, to be highly expressed in various cancers, it was initially discovered to be expressed in macrophages. The recent studies have demonstrated that the expression of PD-L2 correlates with adverse prognosis in a variety of neoplasms, including gastric and colorectal cancers.^28^ PD-L2 is further upregulated on tumor-associated macrophages (TAMs) which are one of the most abundant immune cells infiltrating the TME. TAMs are typically classified into M1 and M2 based on their distinct functional phenotypes. M1 TAMs exerted proinflammatory and anti-tumor activities in cancer patients by stimulating T cells to generate Th1/cytotoxic responses and releasing nitrogen oxide (NO), whereas M2-like TAMs promote tumor growth and tumor immune escape. The expression of PD-L2 was shown to correlate significantly and positively with macrophage infiltration.^29^ Activated macrophages secrete cytokines, including IL-6, TNFα, and TGF-β1, which can cause cholangiocyte proliferation, fibrosis, and cholangiocarcinoma development. In addition, TAMs affect the prognosis of CCA patients by inhibiting the anti-tumor function of T cells, inducing tumor angiogenesis, and releasing Wnt3a and Wnt7b to promote CCA cell proliferation.^30^

Also, in line with this argumentation regarding pCCA is the observation that CD4+cells were downregulated in the tumor compartment of pCCA patients as an abundance of CD4+cells is associated with better oncological outcome.^31^

While not the primary aim of our study, survival analysis was conducted for our study cohort. In terms of immune cells and co-expression of checkpoints, CD4+PD1+ in the tumor were prognostic for reduced CCS in iCCA. PD-1 is a T cell surface receptor that binds with programmed cell death ligand-1 (PD-L1) on tumor cells. The PD-1/PD-L1 axis serves as a negative modulator of the immune system, when binding together, inhibiting T cell-mediated cytotoxicity. As overexpression of PD-1/PD-L1 contributes to establishing an immunosuppressive tumor, it is commonly associated with tumor progression and metastasis.^32^ Interestingly, pCCA CD8+TIGIT+cells showed borderline significance for a slightly protective hazard regarding RFS and CCS. While TIGIT is common to be adversely associated with oncological prognosis, TIGIT is usually highly expressed in specialized CD4+cells and lowly expressed on CD4+ and CD8+ exhausted T cells.^33^ Also, there is further evidence that TIGIT+CD8+ T cells do not necessarily represent a state of immune exhaustion explaining this observation.^34^

### Clinical Implications

In CCA, the TME plays a central role in driving tumor progression, immune evasion, and therapeutic resistance. The TME comprises not only tumor cells but also various non-malignant cell populations, including immune cells, fibroblasts, endothelial cells, as well as diverse signaling molecules, components of the extracellular matrix (ECM), and vasculature. These elements interact to create a complex environment that supports tumor growth, angiogenesis, and immune suppression.^35^ The TME in CCA is typically characterized by dense desmoplastic stroma and a high presence of cancer-associated fibroblasts (CAFs) and tumor-associated macrophages (TAMs). CAFs contribute to tumor progression by secreting factors that promote ECM remodeling, angiogenesis, and immune suppression.^4^ TAMs are often skewed towards an anti-inflammatory M2 phenotype, which fosters tumor growth, tissue remodeling, and suppression of immune responses.^28^ Conversely, M1 TAMs play a proinflammatory role, exerting anti-tumor activity by stimulating T cells and releasing cytokines such as nitric oxide.^36^ Among immune cells, T cells—specifically CD8+ cytotoxic T lymphocytes (CTLs) and CD4+ helper T cells—are critical in the TME. Under normal circumstances, CD8+ T cells mediate direct tumor cell destruction. However, in the immunosuppressive TME, immune checkpoints such as TIM-3 and PD-1 are often upregulated on CD8+ T cells, leading to functional exhaustion and impaired cytotoxicity.^27^ This immune checkpoint-mediated suppression facilitates tumor immune evasion and serves as a primary target for immune checkpoint inhibitors (ICIs), including PD-1/PD-L1 inhibitors and emerging anti-TIM-3 therapies.^37^

Our research indicates a strong correlation between distinct immune cell infiltrates in the TME and various BC parameters. As especially co-stimulatory signals are nowadays targetable by specific immune-based drugs, BC might be a parameter used for sophisticated patient selection. Especially myosteatosis and obesity has shown notable effects on immune cells within the tumor compartment in CCA patients in our analysis. In iCCA, patients with myosteatosis showed less expression of TIM-3+CD8+cells while PD-1+TIM-3+CD4+cells were more likely to be expressed in obese patients. Studies suggest that blocking PD-1/PD-L1 may alleviate the inhibitory effect on T cells.^38^ Clinical trials in patients with melanoma, colorectal cancer, prostate cancer, non-small cell lung cancer and renal cell carcinoma have confirmed the benefits of PD-1/PD-L1 blockade and have transformed the treatment approach for these cancer patients. Recent research has shown that ICIs is a promising new approach to treating CCA, with encouraging results.^39^ Thus, obese patients might be more prone to effective treatment with immune checkpoint inhibitors (ICIs). These therapies can alleviate the inhibitory effect of the PD-1/PD-L1 axis on T cells, thereby restoring their cytotoxic activity. In contrast, patients with myosteatosis exhibit a reduction in TIM-3+CD8+ cells within tumor tissues, which may indicate a poorer response to ICIs. Interestingly, a similar observation was recently done for HCC in which patients with myosteatosis did not respond well to anti-PD1 immunotherapy.^8^ Similarly, myosteatotic patients might be less prone to treatment response to anti-TIM-3 drugs which are currently under investigation.^40^ In summary, the findings of this study suggest that body composition may serve as a crucial parameter in predicting the response to ICIs, particularly in cases where the infiltration of distinct immune cell subsets is closely associated with changes in body composition.

On a theoretical basis, the same accounts for our observation in pCCA in which the expression levels of PD-L2+CD68+ macrophages were elevated in patients with myosteatosis. Functionally re-polarizing these M2 TAMs into M1 Tams with anti-tumor activity have recently been a research focus. Progress has been made for baicalin, 8-bromo-7-methoxychrysin (BrMC), and colony-stimulating factor-1 receptor CSF-1R blockers in promoting the conversion of the macrophage phenotype.^41^ As PD-L2+CD68+macrophages were enriched in myosteatotic patients, these individuals might be more interesting for these novel treatment approaches.

Given the prevalence of changes in BC in CCA and the corresponding changes in immune cell profiles, we advocate a proactive management of these BC shifts. Sarcopenia, sarcopenic obesity, and myosteatosis are all characterized by a shared clinical feature—a decline in muscular function. Empirical evidence underlines the supremacy of exercise to train muscular function and augment overall health status.^42^ However, a minimum duration of three months or longer may be necessitated to elicit significant enhancement in pertinent clinical indices. As upfront surgery without neoadjuvant treatment is currently the standard of care in CCA, a prolonged preoperative intervention might not be feasible in these individuals especially if preoperative hospitalization due to infections especially in pCCA is often necessary. Dietary interventions are likely the most effective approach to correct alterations in BC. It is strongly advised to optimize nutritional intake, ensuring the adequacy of protein and other vital nutrients. In addition, dietary supplements such as creatine, fish oil, and vitamins possess the potential to promote an increase in skeletal muscle mass. Also, interventions regarding immunonutrition or defined supplements might be useful to improve the preoperative condition. Of note, in a clinical model of colorectal cancer, the agents eicosapentaenoic acid and docosahexaenoic acid (DHA) have already demonstrated efficacy in circumventing tumor-associated myosteatosis.^43^

### Biological and Genetic Differences of iCCA and pCCA

The decision to analyzse iCCA and pCCA separately was driven by well-established biological and genetic distinctions between these subtypes. ICCA arises from the bile ducts within the liver, and its TME is characterized by a high degree of immune infiltration, often including tumor-associated macrophages (TAMs) and T cell exhaustion markers. In contrast, pCCA originates near the bile duct hilum, where it interacts more directly with the biliary epithelium, resulting in distinct stromal features and patterns of immune evasion.^35^

On a genetic level, iCCA and pCCA exhibit differences in their mutational landscapes. The iCCA frequently harbors mutations in genes such as IDH1/2, FGFR2, and KRAS, which are less common in pCCA, where alterations in genes like TP53 and SMAD4 are more prevalent.^44^ These genetic variations contribute to distinct tumor behaviors, including differences in metastatic potential, response to chemotherapy, and susceptibility to targeted therapies.

These biological and genetic differences justify the separate analysis of iCCA and pCCA as pooling the subtypes could obscure key immune and tumor characteristics that are critical for understanding the disease and developing subtype-specific therapeutic strategies. For example, the differential expression of immune checkpoints such as PD-L1 and TIM-3 in the two subtypes highlights the need for tailored immunotherapeutic approaches.^45^ Therefore, by analyzing iCCA and pCCA separately, we aimed to provide a more precise understanding of the immune dynamics within each subtype, supporting the potential for personalized treatment strategies.

### Limitations

Our multiplexed immunofluorescence (mIF) approach provided valuable insights into immune cell populations within the CCA tumor microenvironment. However, certain limitations should be acknowledged. While we focused on PD-1 and TIM-3 as key markers of immune exhaustion, additional markers such as LAG-3 and CTLA-4 were not included that may limit the full characterization of T cell dysfunction. Future studies could integrate these markers to offer a more comprehensive immune profile.^35^ Furthermore, CD68, though commonly used to identify macrophages, does not distinguish between the M1 and M2 phenotypes, which are functionally distinct in the tumor microenvironment. Incorporating markers such as CD163 could provide more detailed insights into macrophage roles in tumor progression. Finally, while mIF is sensitive in detecting multiple markers, it may introduce variability in detecting low-expressing markers. Future studies could complement this technique with other methods, such as single-cell sequencing, to validate and extend these findings.

As in the nature of single-center studies, our data have a limited sample size which is also explained by the complex method and naturally warrants further validation by other datasets or multicentric approaches. These larger data sets would provide more diverse data, support generalizability and validate our findings. Further studies should also incorporate longitudinal data to examine changes of BC over time and the influence of BC-associated immune cell characteristics on disease progression and treatment response. Also, further studies focusing on potential contributing factors e.g. dietary factors or genetic characteristics should be in the center of interest as these factors might be associated with our findings and provide a more nuanced understanding on the observed relationships.

We further included a large dataset within our univariate and multivariate analysis, which might be prone for confounding variables. However, as this is the first structural investigation between BC and immune cells in CCA, we decided for a holistic approach to determine important associations which have to be reassured by further research. While not the primary aim of our research, we also assessed survival in our cohort by means of Kaplan–Meier analysis and multivariate Cox regressions. Due to the limited sample size, no further prediction models were feasible within our dataset.

To the best of our knowledge, we provide the first systematic analysis of the association of BC and clinically relevant immune cell subsets in cholangiocarcinoma. Our data show a strong association between various BC parameters and distinct immune cells within the tumor itself as well as within the normal parenchyma. Not only did these immune cell infiltrates show prognostic value, different immune cell characteristics of patient with impaired BC also suggest a different response to modern immunotherapies.

## Credit authorship contribution statement

Conceptualization, G.W and J.B.; methodology, G.W and J.B.; software, G.W and J.B.; validation, D.L and, T.M.; formal analysis, U.P.N.; investigation, J.S, S.L and S.O.; resources, G.W.; data curation, G.W, D.L and J.B.; writing—original draft preparation, G.W.; writing—review and editing, G.W, D.L, T.M, C.O, J.S, S.L, T.U,S.O, T.L, E.D, U.P.N, L.H and J.B.; visualization, J.B.; supervision, U.P.N.; project administration, J.B.; funding acquisition, G.W and D.L. All authors have read and agreed to the published version of the manuscript.

## Funding

Guanwu Wang was funded by China Scholarship Council (Grant number: 202108430018). Dong Liu was funded by China Scholarship Council (Grant number: 202208080011).

## Consent for publication

Not applicable as no identifying material is published.

## Availability of data and materials

The data presented in this study are available on request from the corresponding author.

## Declaration of competing interest

All authors disclosed no relevant relationships.
