# Supplementary figures and images for: Body Composition in Cholangiocarcinoma Affects Immune Cell Populations in the Tumor and Normal Liver Parenchyma
Source: J Clin Exp Hepatol. 2024 Nov 26;15(2):102460. doi: 10.1016/j.jceh.2024.102460 (PMC11697564; doi:10.1016/j.jceh.2024.102460)

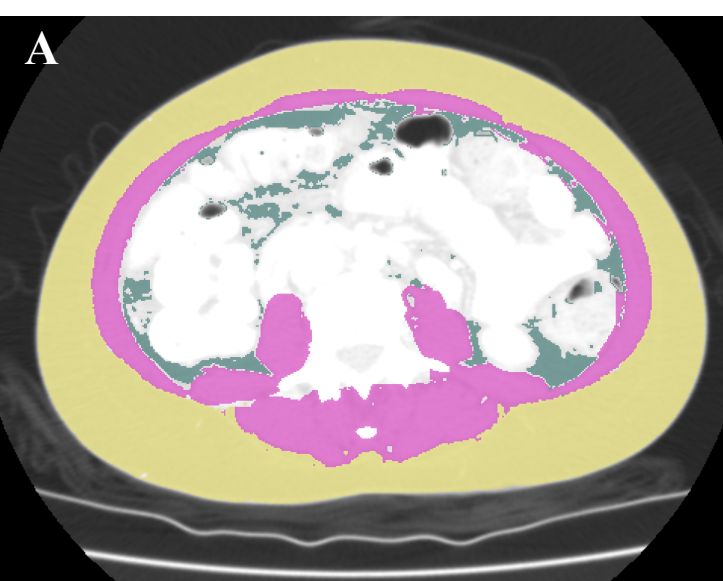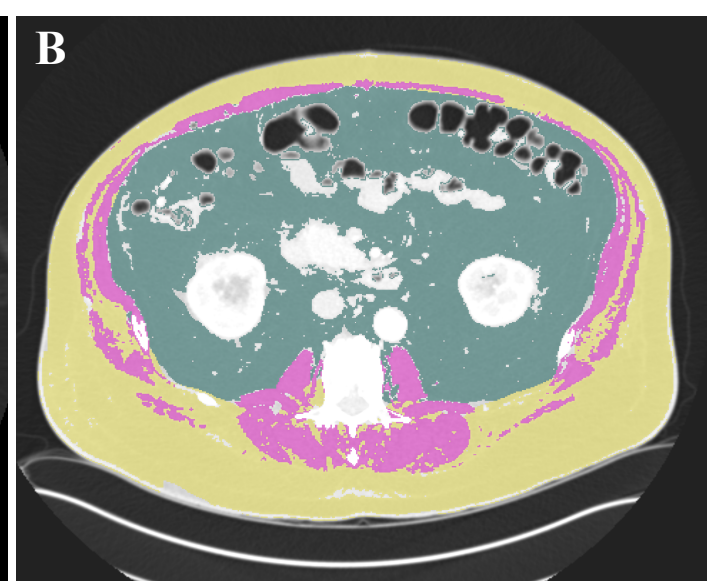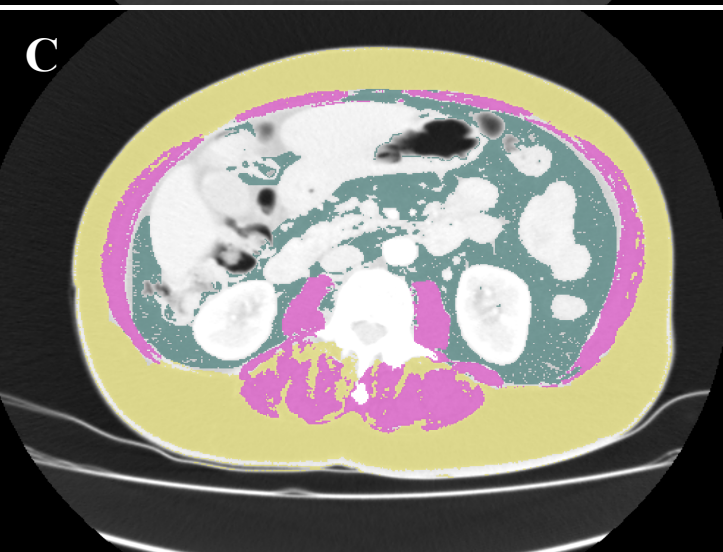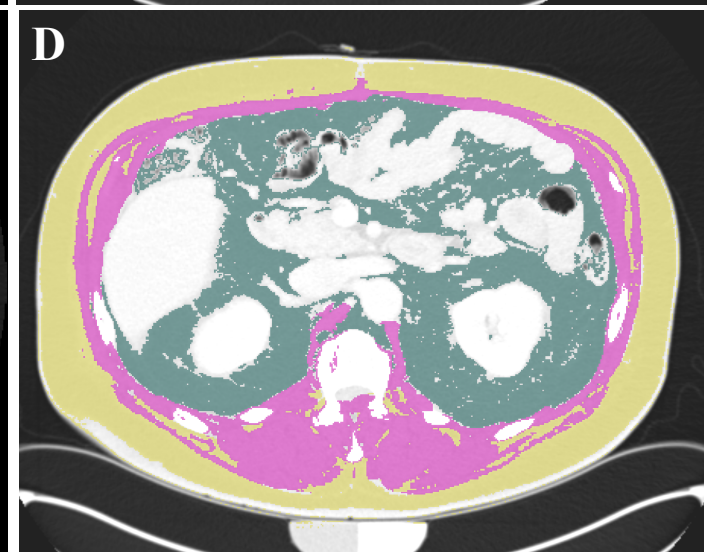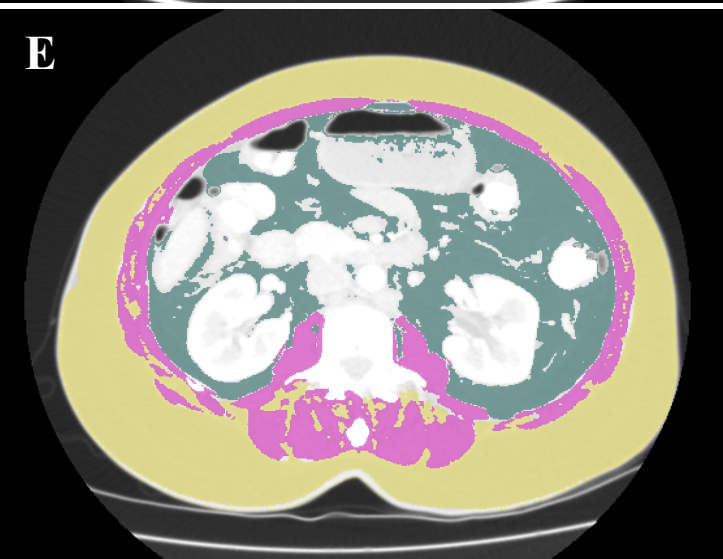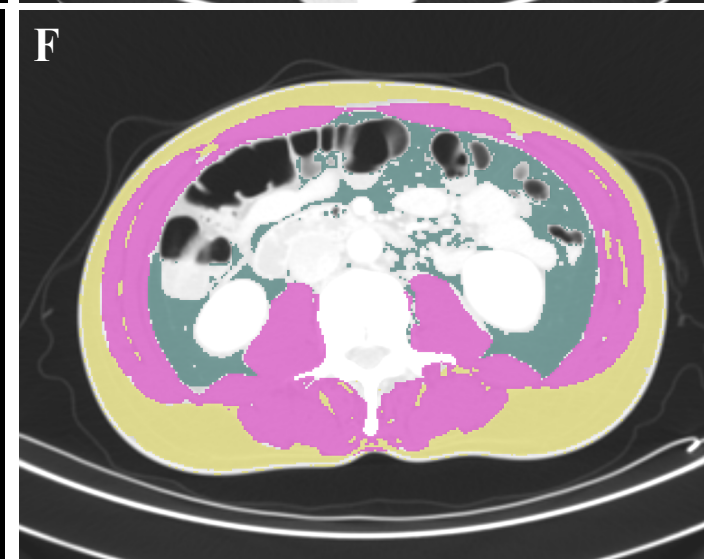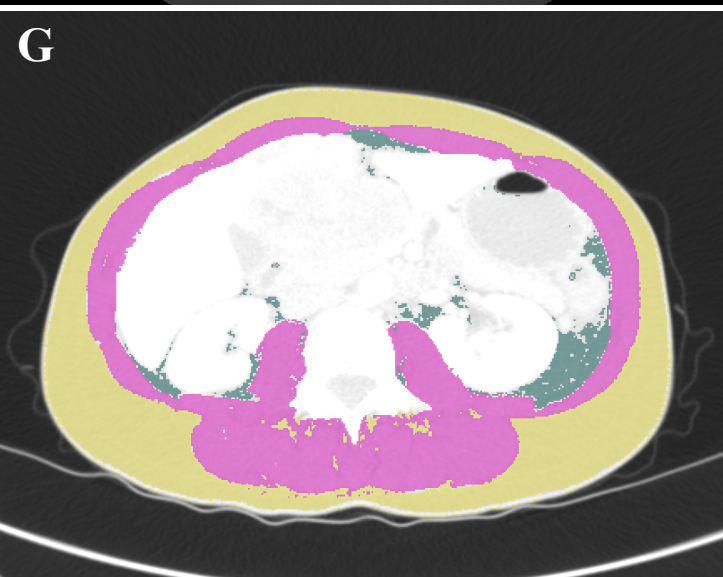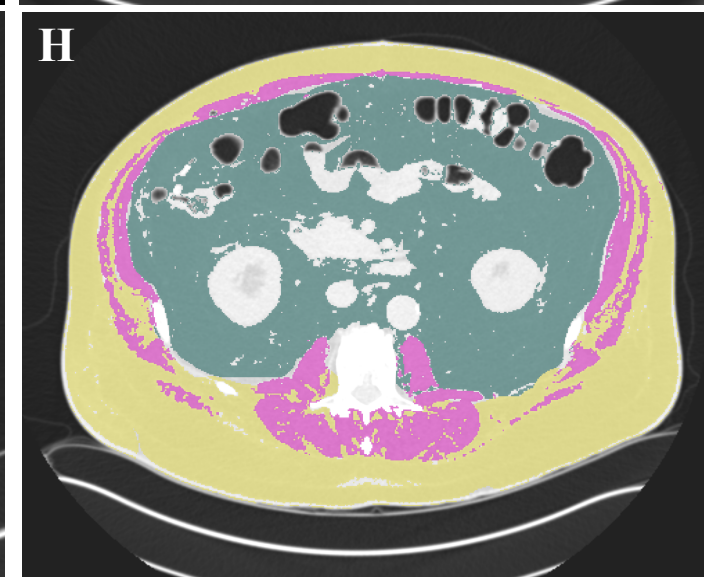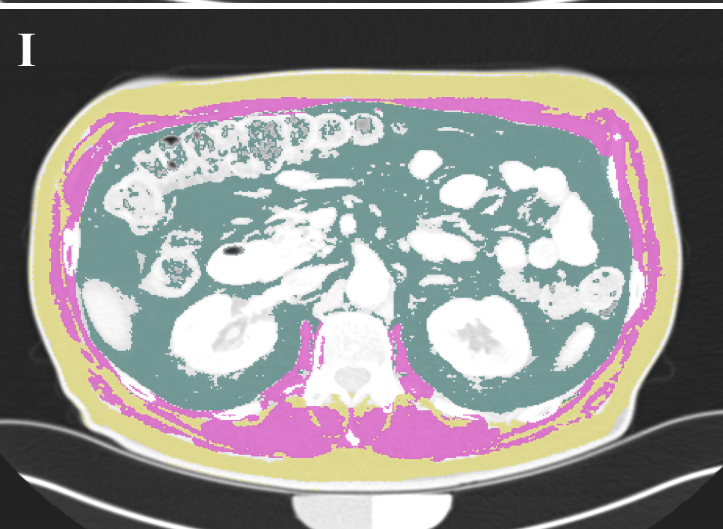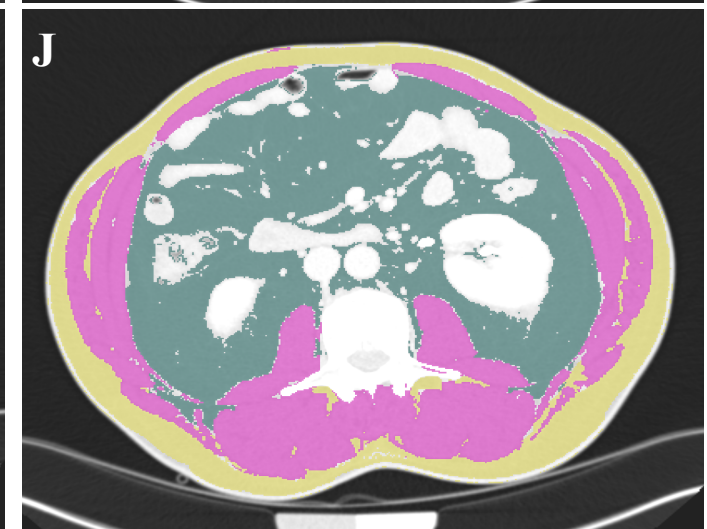

Supplement: Multimedia component 1 [file mmc1.pdf]
