# Supplementary material for: Body Composition in Cholangiocarcinoma Affects Immune Cell Populations in the Tumor and Normal Liver Parenchyma
Source: J Clin Exp Hepatol. 2024 Nov 26;15(2):102460. doi: 10.1016/j.jceh.2024.102460 (PMC11697564; doi:10.1016/j.jceh.2024.102460)
Supplement: Multimedia component 2 [file mmc2.docx]

** Supplementary Figure 2: Survival analysis**

Patients with iCCA displayed a median RFS of 8 months and a median CCS of 30 months (A+B). Patients with pCCA displayed a median RFS of 29 months and a median CCS of 28 months (C+D).
