# Supplementary material for: Body Composition in Cholangiocarcinoma Affects Immune Cell Populations in the Tumor and Normal Liver Parenchyma
Source: J Clin Exp Hepatol. 2024 Nov 26;15(2):102460. doi: 10.1016/j.jceh.2024.102460 (PMC11697564; doi:10.1016/j.jceh.2024.102460)
Supplement: Multimedia component 3 [file mmc3.docx]

**Table S1** Univariate Analysis and Multivariate Analysis BC with Multiplex Data in Intrahepatic CCA (n=48)

| **Outcome** | **Desriptives** | | **Univarite analysis** | | **Multivariate Analysis** | |
| --- | --- | --- | --- | --- | --- | --- |
| **BMI** | **＜25(n=25）** | **≥25(n=23)** | **OR (95% CI)** | **p=** | **OR (95% CI)** | **p=** |
| Sex  (male/female(%); ref=male) | 11(44.0)/14(56.0) | 12(52.2)/11(45.8) | 0.720(0.231-2.245） | 0.572 |  |  |
| Age  (≤65/ >65 years; ref=≤65) | 68(56-73) | 66(56-71) | 0.720(0.231-2.245） | 0.572 |  |  |
| PVE  (No/Yes(%);ref=No) | 25(100.0)/0(0) | 20(80.7)/3(13.0) | n.a | 0.999 |  |  |
| ASA  ((I/II)/(III/IV) (%);ref= I/II) | 15(60.0)/10(40.0) | 9(39.1)/14(60.9) | 2.333(0.733-7.430) | 0.152 |  |  |
| Neoadjuvant therapy  ((No/Yes(%);ref=No) | 24(96.0)/1(4.0) | 21(91.3)/2(8.7) | 2.286(0.193-27.046) | 0.512 |  |  |
| AST  U/L(≤40/ >40; ref=≤40) | 38.0(25.3-53.5) | 39.5(26.5-61.25) | 0.993(0.320-3.085) | 0.990 |  |  |
| ALT  U/L(≤40/ >40; ref=≤40) | 22.0(16.0-72.0) | 30.5(23.5-45.8) | 0.679(0.211-2.179) | 0.515 |  |  |
| GGT  U/L(≤100/ >100; ref=≤100) | 116.0(69.5-318.0) | 158.0(67.0-616.0) | 1.750(0.524-5.842) | 0.363 |  |  |
| Bilirubin  mg/dl(≤1/ >1; ref=≤1) | 0.7(0.4-1.3) | 0.5(0.4-0.9) | 0.667(0.161-2.769) | 0.577 |  |  |
| Platelet count  (≤250/ >250; ref=≤250) | 266(215-340) | 227(189-308) | 0.705(0.221-2.253) | 0.555 |  |  |
| Prothrombin time  (≤110/ >110; ref=≤110) | 100(95-111) | 97(84-104) | 0.500(0.108-2.314) | 0.375 |  |  |
| INR  (≤1/>1; ref=≤1) | 1.00(0.95-1.03) | 1.02(0.95-1.11) | 1.867(0.570-6.109) | 0.302 |  |  |
| Hemoglobin  g/L(≤13/ >13; ref=≤13) | 13(12-14) | 14(12-15) | 2.450(0.746-8.043) | 0.140 |  |  |
| CRP  mg/L(≤10/ >10; ref=≤10) | 13.5(5.1-33.5) | 9.4(3.4-17.0) | 1.100(0.332-3.640) | 0.876 |  |  |
| Operative time  minutes(≤360/ >360; ref=≤360) | 283(226-345) | 290(230-359) | 2.037(0.428-9.704) | 0.372 |  |  |
| Intraop PRBC  (No/Yes;ref=No) | 13(52.0)/12(48.0) | 15(65.2)/8(34.8) | 0.578(0.181-1.848) | 0.355 |  |  |
| Intraop FFP  ((No/Yes(%);ref=No) | 14(56.0)/11(44.0) | 12(52.2)/11(47.8) | 1.167(0.374-3.637) | 0.790 |  |  |
| R1 resection  (R0/R1) (%); ref= R0) | 22(88.0)/3(12.0) | 22(95.7)/1(4.3) | 0.333(0.032-3.457) | 0.357 |  |  |
| MVI  (No/Yes(%);ref=No) | 15(60.0)/10(40.0) | 15(65.2)/8(34.8) | 0.800(0.247-2.586) | 0.709 |  |  |
| LVI  (No/Yes(%);ref=No) | 23(92.0)/2(8.0) | 13(65.5)/7(30.4) | 6.192(1.117-34.316) | **0.037** | 14.990(1.657-135.594) | **0.016** |
| Tumor grading  ((G1/G2)/( G3/G4) (%);ref= G1/G2) | 19(76.0)/4(16.0) | 16(69.6)/6(26.1) | 1.781(0.427-7.438) | 0.429 |  |  |
| pN category  (N0/N1(%);ref=N0) | 19(76.0)/5(20.0) | 12(52.2)/10(43.0) | 3.167(0.868-11.551) | 0.081 |  |  |
| ICU time  days(≤1＞1(%),ref=1） | 16(64.0)/9(36.0) | 17(73.9)/6(26.1) | 0.627(0.182-2.164) | 0.461 |  |  |
| Hospitalization  days(≤14/>14(%); ref=≤14) | 16(9-25) | 13(9-18) | 0.356(0.110-1.149) | 0.084 |  |  |
| Adjuvant therapy  (No/Yes(%);ref=No) | 19(76.0)/6(24.0) | 17(73.9)/6(26.1) | 1.118(0.302-4.130) | 0.868 |  |  |
| Tumor CD4 PD-1 grp  (grouped by median, ref=low expression) | 72.52 (7.82-376.98) | 265.43 (125.53-1650.78) | 4.148(1.179-14.589) | **0.027** | 2.933(0.480-17.930) | 0.244 |
| Tumor CD4 PD-1 LAG-3 TIM-3 grp  (grouped by median, ref=low expression) | 1.54 (0-22.362) | 28.15 (4.27-285.49) | 4.148(1.179-14.589) | **0.027** | 0.940(0.030-29.849) | 0.972 |
| Tumor CD4 PD-1 TIM-3 grp  (grouped by median, ref=low expression) | 5.06 (0-99.83) | 164.25 (28.34-815.10) | 4.148(1.179-14.589) | **0.027** | 8.904(1.615-49.088) | **0.012** |
| Tumor CD4 LAG3 TIM-3 grp  (grouped by median, ref=low expression) | 7.20 (1.41-55.62) | 60.72 (22.19-334.85) | 4.148(1.179-14.589) | **0.027** | 1.193(0.154-9.217) | 0.866 |
| Tumor CD8 PD-1 TIM-3 grp  (grouped by median, ref=low expression) | 15.91 (2.59-61.59) | 109.20 (25.80-1288.38) | 4.148(1.179-14.589) | **0.027** | 2.418(0.526-11.105) | 0.256 |
| **Sarcopenia** | **No****(n=14)** | **Yes(n=34)** | **OR (95% CI)** | **p=** | **OR (95% CI)** | **p=** |
| Sex  (male/female(%); ref=male) | 10(71.4)/4(28.6) | 13(38.2)/21(61.8) | 4.038(1.047-15.581) | **0.043** | 4.038(1.047-15.581) | **0.043** |
| Age  (≤65/ >65 years; ref=≤65) | 61(58-69) | 69(55-73) | 1.689(0.481-5.933) | 0.414 |  |  |
| PVE  (No/Yes(%);ref=No) | 14(100.0)/0(0) | 31(91.2)/3(8.8) | --- | 0.999 |  |  |
| ASA  ((I/II)/(III/IV) (%);ref= I/II) | 6(42.9)/8(57.1) | 18(52.9)/16(47.1) | 0.667(0.190-2.338) | 0.526 |  |  |
| Neoadjuvant therapy  ((No/Yes(%);ref=No) | 12(85.7)/2(14.3) | 33(97.1)/1(2.9) | 0.182(0.015-2.192) | 0.180 |  |  |
| AST  U/L(≤40/ >40; ref=≤40) | 37.0(25.0-44.0) | 41.0(26.0-60.0) | 2.025(0.561-7.312) | 0.281 |  |  |
| ALT  U/L(≤40/ >40; ref=≤40) | 24.5(20.3-50.8) | 29(19.0-72.5) | 1.974(0.515-7.558) | 0.321 |  |  |
| GGT  U/L(≤100/ >100; ref=≤100) | 111.0(69.5-198.5) | 184.0(67.0-616.0) | 0.759(0.202-2.848) | 0.683 |  |  |
| Bilirubin  mg/dl(≤1/ >1; ref=≤1) | 0.5(0.4-0.7) | 0.6(0.4-1.2) | 4.500(0.509-39.773) | 0.176 |  |  |
| Platelet count  (≤250/ >250; ref=≤250) | 231(211-352) | 266(189-315) | 1.920(0.518-7.121) | 0.329 |  |  |
| Prothrombin time  (≤110/ >110; ref=≤110) | 100(86-107) | 99(92-108) | 1.540(0.275-8.635) | 0.624 |  |  |
| INR  (≤1/>1; ref=≤1) | 0.98(0.96-1.10) | 1.01(0.95-1.05) | 1.167(0.321-4.247) | 0.815 |  |  |
| Hemoglobin  g/L(≤13/ >13; ref=≤13) | 231(211-352） | 266(189-315) | 0.370(0.095-1.447) | 0.153 |  |  |
| CRP  mg/L(≤10/ >10; ref=≤10) | 9.0(4.9-25.7) | 10.5(4.2-28.5) | 1.167(0.317-4.299) | 0.817 |  |  |
| Operative time  minutes(≤360/ >360; ref=≤360) | 278(214-345) | 286(230-358) | 1.286(0.226-7.306) | 0.777 |  |  |
| Intraop PRBC  (No/Yes;ref=No) | 9(64.3)/5(35.7) | 19(55.9)/15(44.1) | 1.421(0.393-5.141) | 0.592 |  |  |
| Intraop FFP  ((No/Yes(%);ref=No) | 8(57.1)/6(42.9) | 18(52.9)/16(47.1) | 0.368(0.066-2.050) | 0.791 |  |  |
| R1 resection  (R0/R1) (%); ref= R0) | 13(92.9)/1(7.1) | 31(91.2)/3(8.8) | 1.258(0.120-12.245) | 0.848 |  |  |
| MVI  (No/Yes(%);ref=No) | 11(78.6)/3(21.4) | 19(55.9)/15(44.1) | 2.895(0.682-12.279) | 0.149 |  |  |
| LVI  (No/Yes(%);ref=No) | 12(85.7)/2(14.3) | 24(70.6)/7(20.6) | 1.750(0.314-9.748) | 0.523 |  |  |
| Tumor grading  ((G1/G2)/( G3/G4) (%);ref= G1/G2) | 11(78.6)/2(14.3) | 24(70.6)/8(23.5) | 1.833(0.333-10.095) | 0.486 |  |  |
| pN category  (N0/N1(%);ref=N0) | 10(71.4)/3(21.4) | 21(61.8)/12(35.3) | 1.905(0.437-8.303) | 0.391 |  |  |
| ICU time  days(≤1＞1(%),ref=1） | 10(71.4)/4(28.6) | 23(67.6)/11(32.4) | 1.196(0.306-4.678) | 0.797 |  |  |
| Hospitalization  days(≤14/>14(%); ref=≤14) | 10(7-26) | 15(10-24) | 2.025(0.561-7.312) | 0.218 |  |  |
| Adjuvant therapy  (No/Yes(%);ref=No) | 13(92.9)/1(7.1) | 23(67.6)/11(32.4) | 0.982(0.268-3.602) | 0.978 |  |  |
| **Myosteatosis** | **No(n=20)** | **Yes(n=28)** | **OR (95% CI)** | **p=** | **OR (95% CI)** | **p=** |
| Sex  (male/female(%); ref=male) | 13(65.0)/7(35.0) | 10(35.7)/18(64.3) | 3.343(1.006-11.107) | **0.049** | 2.587(0.437-15.312) | 0.295 |
| Age  (≤65/ >65 years; ref=≤65) | 60(53-71) | 69(59-74) | 1.630(0.513-5.177) | 0.408 |  |  |
| PVE  (No/Yes(%);ref=No) | 18(90.0)/2(10.0) | 27(96.4)/1(3.6) | 0.333(0.028-3.954) | 0.384 |  |  |
| ASA  ((I/II)/(III/IV) (%);ref= I/II) | 9(45.0)/11(55.0) | 15(53.6)/13(46.4) | 0.709(0.224-2.244) | 0.559 |  |  |
| Neoadjuvant therapy  ((No/Yes(%);ref=No) | 19(95.0)/1(5.0) | 26(92.9)/2(7.1) | 1.462(0.123-17.318) | 0.764 |  |  |
| AST  U/L(≤40/ >40; ref=≤40) | 42.0(30.0-54.8) | 31.0(24.3-58.3) | 0.431(0.134-1.394) | 0.160 |  |  |
| ALT  U/L(≤40/ >40; ref=≤40) | 38.0(25.0-73.0) | 21.5(14.8-63.0) | 0.679(0.210-2.192) | 0.517 |  |  |
| GGT  U/L(≤100/ >100; ref=≤100) | 188.0(109.0-270.0) | 83.0(62.5-543.5) | 0.178(0.046-0.694) | **0.013** | 0.182(0.028-1.171) | 0.073 |
| Bilirubin  mg/dl(≤1/ >1; ref=≤1) | 0.6(0.4-0.9) | 0.5(0.4-1.2) | 1.867(0.415-8.398) | 0.416 |  |  |
| Platelet count  (≤250/ >250; ref=≤250) | 241(212-306) | 266(189-340) | 1.719(0.525-5.625) | 0.371 |  |  |
| Prothrombin time  (≤110/ >110; ref=≤110) | 99(85-108) | 99(94-106) | 1.429(0.307-6.638) | 0.649 |  |  |
| INR  (≤1/>1; ref=≤1) | 1.00(0.95-1.11) | 1.00(0.95-1.03) | 0.929(0.282-3.062) | 0.903 |  |  |
| Hemoglobin  g/L(≤13/ >13; ref=≤13) | 14.0(12.2-14.8) | 12.4(12.0-14.2) | 0.210(0.058-0.760) | **0.017** | 0.104(0.016-0.691) | **0.019** |
| CRP  mg/L(≤10/ >10; ref=≤10) | 14.4(7.0-29.3) | 7.0(2.9-29.4) | 0.281(0.079-1.007) | 0.051 |  |  |
| Operative time  minutes(≤360/ >360; ref=≤360) | 290(226-358) | 286(230-353) | 0.667(0.145-3.059) | 0.602 |  |  |
| Intraop PRBC  (No/Yes;ref=No) | 13(65.0)/7(35.0) | 15(53.6)/13(35.0) | 1.610(0.494-5.246) | 0.430 |  |  |
| Intraop FFP  ((No/Yes(%);ref=No) | 10(50.0)/10(50.0) | 16(57.1)/12(42.9) | 0.750(0.237-2.375) | 0.625 |  |  |
| R1 resection  (R0/R1) (%); ref= R0) | 18(90.0)/2(10.0) | 26(92.9)/2(7.1) | 0.692 (0.785-30.289) | 0.725 |  |  |
| MVI  (No/Yes(%);ref=No) | 13(65.0)/7(35.0) | 17(60.7)/9(33.3) | 1.202(0.365-3.956) | 0.762 |  |  |
| LVI  (No/Yes(%);ref=No) | 15(75.0)/3(15.0) | 21(75.0)/6(21.4) | 1.429(0.307-6.638) | 0.649 |  |  |
| Tumor grading  ((G1/G2)/( G3/G4) (%);ref= G1/G2) | 15(75.0)/4(20.0) | 20(71.4)/6(21.4) | 1.125(0.269-4.707) | 0.872 |  |  |
| pN category  (N0/N1(%);ref=N0) | 12(60.0)/8(40.0) | 19(67.9)/7(25.0) | 0.553(0.159-1.920) | 0.351 |  |  |
| ICU time  days(≤1＞1(%),ref=1） | 15(75.0)/5(25.0) | 18(64.3)/10(35.7) | 1.667(0.466-5.956) | 0.432 |  |  |
| Hospitalization  days(≤14/>14(%); ref=≤14) | 14(9-24) | 14(9-26) | 1.222(0.387-3.864) | 0.733 |  |  |
| Adjuvant therapy  (No/Yes(%);ref=No) | 15(75.0)/5(25.0) | 21(75.0)/7(25.0) | 1(0.266-3.763) | 1 |  |  |
| Tumor CD4 PD-1 LAG-3 grp  (grouped by median, ref=low expression) | 125.53 (15.207-951.33) | 11.57 (0-40.58) | 0.226(0.062-0.825) | **0.024** | 1.114(0.121-10.234) | 0.924 |
| Tumor CD8 PD-1 grp  (grouped by median, ref=low expression) | 837.17 (242.92-2066.75) | 212.30 (425.66-518.91) | 0.226(0.062-0.825) | **0.024** | 0.519(0.072-3.748) | 0.515 |
| Tumor CD8 LAG-3 grp  (grouped by median, ref=low expression) | 1229.19 (406.82-3012.64) | 336.65 (80.36-647.00) | 0.226(0.062-0.825) | **0.024** | 0.289(0.053-1.579) | 0.152 |
| Tumor CD8 TIM-3 grp  (grouped by median, ref=low expression) | 369.26 (223.21-2264.68) | 125.41 (21.02-456.17) | 0.143(0.036-0.562) | **0.005** | 0.118(0.019-0.747) | **0.023** |
| **VFA** | **≤100(n=22)** | **＞100(n=26)** | **OR (95% CI)** | **p=** | **OR (95% CI)** | **p=** |
| Sex  (male/female(%); ref=male) | 5(22.7)/17(77.3) | 18(69.2)/8(30.8) | 0.131(0.036-0.479) | **0.002** | 0.070(0.006-0.886) | **0.040** |
| Age  (≤65/ >65 years; ref=≤65) | 63(54-74) | 67(58-71) | 1.636(0.521-25.670) | 0.399 |  |  |
| PVE  (No/Yes(%);ref=No) | 20(90.9)/2(9.1) | 25(96.2)/1(3.8) | 0.400(0.034-4.736) | 0.467 |  |  |
| ASA  ((I/II)/(III/IV) (%);ref= I/II) | 12(54.5)/10(45.5) | 12(46.2)/14(53.8) | 1.400(0.448-4.376) | 0.563 |  |  |
| Neoadjuvant therapy  ((No/Yes(%);ref=No) | 21(95.5)/1(4.5) | 24(92.3)/2(7.7) | 1.750(0.148-20.707) | 0.657 |  |  |
| AST  U/L(≤40/ >40; ref=≤40) | 41.5(24.3-53.8) | 37.0(27.5-56.8) | 0.611(0.195-1.919) | 0.399 |  |  |
| ALT  U/L(≤40/ >40; ref=≤40) | 26.0(18.5-70.0) | 28.0(21.0-52.0) | 0.635(0.198-2.037) | 0.445 |  |  |
| GGT  U/L(≤100/ >100; ref=≤100) | 101.5(65.0-440.3) | 157.0(70.8-370.8) | 1.600(0.474-5.396) | 0.449 |  |  |
| Bilirubin  mg/dl(≤1/ >1; ref=≤1) | 0.5(0.4-1.2) | 0.6(0.4-0.9) | 0.714(0.175-2.913) | 0.639 |  |  |
| Platelet count  (≤250/ >250; ref=≤250) | 288(209-389) | 229(194-301) | 0.489(0.149-1.600) | 0.237 |  |  |
| Prothrombin time  (≤110/ >110; ref=≤110) | 100(92-108) | 98(86-106) | 1.00(0.230-4.349) | 1.000 |  |  |
| INR  (≤1/>1; ref=≤1) | 1.00(0.95-1.05) | 1.01(0.94-1.11) | 1.324(0.407-4.308) | 0.641 |  |  |
| Hemoglobin  g/L(≤13/ >13; ref=≤13) | 12.30 (11.93-13.63) | 14.05 (12.28-14.83) | 5.250(1.477-18.660) | **0.010** | 11.011(0.851-142.512) | 0.066 |
| CRP  mg/L(≤10/ >10; ref=≤10) | 14.0(4.6-37.7) | 9.4(4.7-17.0) | 0.629(0.186-2.129) | 0.456 |  |  |
| Operative time  minutes(≤360/ >360; ref=≤360) | 291(229-345) | 273(229-358) | 0.818(0.179-3.739) | 0.796 |  |  |
| Intraop PRBC  (No/Yes;ref=No) | 13(59.1)/9(40.9) | 15(57.7)/11(42.3) | 1.059(0.335-3.353) | 0.922 |  |  |
| Intraop FFP  ((No/Yes(%);ref=No) | 13(59.1)/9(40.9) | 13(50.0)/13(50.0) | 1.444(0.459-4.544) | 0.529 |  |  |
| R1 resection  (R0/R1) (%); ref= R0) | 19(86.4)/3(13.6) | 25(96.2)/1 (3.8) | 0.253(0.024-2.631) | 0.250 |  |  |
| MVI  (No/Yes(%);ref=No) | 13(59.1)/9(40.9) | 17(65.4)/9(34.6) | 0.765(0.237-2.470) | 0.654 |  |  |
| LVI  (No/Yes(%);ref=No) | 18(81.8)/2(9.1) | 18(69.2)/7(26.9) | 3.500(0.638-19.195) | 0.149 |  |  |
| Tumor grading  ((G1/G2)/( G3/G4) (%);ref= G1/G2) | 18(81.8)/2(9.1) | 17(65.4)/8(30.8) | 4.235(0.785-22.846) | 0.093 |  |  |
| pN category  (N0/N1(%);ref=N0) | 17(77.3)/4(18.2) | 14(53.8)/11(42.3) | 3.339(0.870-12.821) | 0.079 |  |  |
| ICU time  days(≤1＞1(%),ref=1） | 14(63.6)/8(36.4) | 19(73.1)/7(26.9) | 0.645(0.189-2.199) | 0.483 |  |  |
| Hospitalization  days(≤14/>14(%); ref=≤14) | 16(10-26) | 12(8-36) | 0.303(0.092-0.991) | **0.048** | 3.438(0.175-109.649) | 0.368 |
| Adjuvant therapy  (No/Yes(%);ref=No) | 17(77.3)/5(22.7) | 19(73.1)/7(26.9) | 1.253(0.334-4.694) | 0.738 |  |  |
| Normal CD68 PD-1 grp  (grouped by median, ref=low expression) | 258.37 (61.11-5052.70) | 32.72 (2.45-306.99) | 0.182(0.035-0.939) | **0.042** | 0.345(0.032-3.816) | 0.388 |
| Normal CD68 PD-1 PD-L1 PD-L2 grp  (grouped by median, ref=low expression) | 0 (0-39.76) | 0 (0-0) | 0.088(0.009-0.893) | **0.040** | 0.008(0-0.323) | **0.010** |
| **Sarcopenic_obesity** | **No(n=39)** | **Yes(n=9)** | **OR (95% CI)** | **p=** | **OR (95% CI)** | **p=** |
| Sex  (male/female(%); ref=male) | 17(43.6)/22(56.4) | 6(66.7)/3(33.3) | 0.386(0.084-1.773) | 0.221 |  |  |
| Age  (≤65/ >65 years; ref=≤65) | 65.0(55.0-72.0) | 70.0(63.0-77.5) | 4.083(0.751-22.193) | 0.103 |  |  |
| PVE  (No/Yes(%);ref=No) | 38(97.4)/1(2.6) | 7(77.8)/2(22.2) | 10.857(0.863-136.596) | 0.065 |  |  |
| ASA  ((I/II)/(III/IV) (%);ref= I/II) | 19(48.7)/20(51.3) | 5(55.6)/4(44.4) | 0.760(0.177-3.263) | 0.712 |  |  |
| Neoadjuvant therapy  ((No/Yes(%);ref=No) | 37(94.9)/2(5.1) | 8(88.9)/1(11.1) | 2.312(0.186-28.717) | 0.514 |  |  |
| AST  U/L(≤40/ >40; ref=≤40) | 39.0(25.0-55.0) | 42.0(28.5-56.0) | 0.842(0.196-3.615) | 0.817 |  |  |
| ALT  U/L(≤40/ >40; ref=≤40) | 25.0(18.0-68.0) | 35.0(23.0-90.0) | 0.720(0.117-4.412) | 0.722 |  |  |
| GGT  U/L(≤100/ >100; ref=≤100) | 116.0(67.0-347.0) | 158.0(79.5-713.0） | 0.533(0.122-2.339) | 0.405 |  |  |
| Bilirubin  mg/dl(≤1/ >1; ref=≤1) | 0.6(0.4-1.2) | 0.5(0.4-0.7) | 0.389(0.043-3.546) | 0.402 |  |  |
| Platelet count  (≤250/ >250; ref=≤250) | 255(211-339) | 283(174-316) | 1.319(0.305-5.706) | 0.711 |  |  |
| Prothrombin time  (≤110/ >110; ref=≤110) | 101(95-108) | 88(83-103) | 0(0-0) | 0.999 |  |  |
| INR  (≤1/>1; ref=≤1) | 1.00(0.95-1.03) | 1.09(0.98-1.13) | 10.267(1.143-92.256) | **0.038** | 4.125(0.374-45.520) | 0.247 |
| Hemoglobin  g/L(≤13/ >13; ref=≤13) | 13(45-14) | 13(12-15) | 0.333(0.061-1.812) | 0.203 |  |  |
| CRP  mg/L(≤10/ >10; ref=≤10) | 9.3(5.0-28.5) | 11.0(2.9-29.9) | 1406(0.321-6.160) | 0.651 |  |  |
| Operative time  minutes(≤360/ >360; ref=≤360) | 280(225-355) | 330(358-354) | 0(0-0) | 0.999 |  |  |
| Intraop PRBC  (No/Yes;ref=No) | 21(53.8)/18(46.2) | 7(77.8)/2(22.2) | 0.333(0.061-1.812) | 0.203 |  |  |
| Intraop FFP  ((No/Yes(%);ref=No) | 20(51.3)/19(48.7) | 6(66.7)/3(33.3) | 0.526(0.115-2.410) | 0.408 |  |  |
| R1 resection  (R0/R1) (%); ref= R0) | 36(92.3)/3(7.7) | 8(88.9)/1(11.1) | 1.500(0.138-16.359) | 0.739 |  |  |
| MVI  (No/Yes(%);ref=No) | 25(64.1)/14(35.9) | 5(55.6)/4(44.4) | 1.429(0.329-6.204) | 0.634 |  |  |
| LVI  (No/Yes(%);ref=No) | 33(84.6)/5(12.8) | 3(33.3)/4(44.4) | 8.800(1.502-51.556) | **0.016** | 11.600(1.659-81.102） | 0.014 |
| Tumor grading  ((G1/G2)/( G3/G4) (%);ref= G1/G2) | 31(79.5)/6(15.4) | 4(44.4)/4(44.4) | 5.167(1.004-26.597) | **0.049** | 3.107(0.372-25.953) | **0.295** |
| pN category  (N0/N1(%);ref=N0) | 26(66.7)/12 (30.8) | 5(55.6)/3(33.3) | 1.300(0.266-6.352) | 0.746 |  |  |
| ICU time  days(≤1＞1(%),ref=1） | 26(66.7)/13(33.3) | 7(77.8)/2(22.2) | 0.571(0.104-3.149) | 0.520 |  |  |
| Hospitalization  days(≤14/>14(%); ref=≤14) | 14(9-24) | 14(10-26) | 0.475(0.104-2.175) | 0.338 |  |  |
| Adjuvant therapy  (No/Yes(%);ref=No) | 29(74.4)/10(25.6) | 7(77.8)/2(22.2) | 0.829 (0.147-4.665) | 0.831 |  |  |

Note: The multiplex data were divided into high and low expression groups based on the median.

Abbreviations: ALT, alanine aminotransferase;ASA,American Society of Anesthesiologists;AST, aspartate aminotransferase; BMI, body mass index; CRP, C-­reactive protein; F, female; FFP,Fresh frozen plasma; GGT,gamma-glutamyl transferase; HR, hazard ratio; iCCA, intrahepatic cholangiocarcinoma; ICU, intensive care unit; INR, international normalized ratio; LVI, lymph vascular invasion; M, male; MVI, microvascular invasion; PRBC, packed red blood cells; PVE, portal vein embolization; RFS, recurrence free survival. Variables displaying a p value < 0.05 in the univariate analysis were transferred into a multivariable logistic regression model.

Note: multiplex date(×10^-5^).
