# Supplementary material for: Body Composition in Cholangiocarcinoma Affects Immune Cell Populations in the Tumor and Normal Liver Parenchyma
Source: J Clin Exp Hepatol. 2024 Nov 26;15(2):102460. doi: 10.1016/j.jceh.2024.102460 (PMC11697564; doi:10.1016/j.jceh.2024.102460)
Supplement: Multimedia component 4 [file mmc4.docx]

**Table S2** Univariate Analysis and Multivariate Analysis BC with Multiplex Data in Perihilar CCA (n=48)

| **Outcome** | **Desriptives** | | **Univariate Analysis** | | **Multivariate Analysis** | |
| --- | --- | --- | --- | --- | --- | --- |
| **BMI(kg/m^2^)** | **＜25(n=26）** | **≥25(n=22)** | **OR(95%Cl)** | **p=** | **OR(95%Cl)** | **p=** |
| Sex  (male/female(%); ref=male) | 16(61.5)/10(38.5) | 13(59.1)/9(40.9) | 1.108(0.347-3.535) | 0.863 |  |  |
| Age  (≤65/ >65 years; ref=≤65) | 68(59-75) | 63(55-71) | 0.433(0.136-1.381) | 0.157 |  |  |
| PVE  (No/Yes(%);ref=No) | 16(61.5)/10(38.5) | 12(54.5)/10(45.5) | 1.333(0.421-4.222) | 0.625 |  |  |
| ASA  ((I/II)/(III/IV) (%);ref= I/II) | 11(42.3)/15(57.7) | 13(59.1)/9(40.9) | 0.508(0.160-1.607) | 0.249 |  |  |
| Neoadjuvant therapy  ((No/Yes(%);ref=No) | 25(96.2)/1(3.8) | 20(90.9)/2(9.1) | 2.500(0.211-29.598) | 0.467 |  |  |
| AST  U/L(≤40/ >40; ref=≤40) | 50.0(37.0-95.0) | 53.0(37.5-90.5) | 1.339(0.405-4.426) | 0.632 |  |  |
| ALT  U/L(≤40/ >40; ref=≤40) | 102.0(64.5-164.5) | 69.0(27.3-243.25) | 0.532(0.158-1.791) | 0.308 |  |  |
| GGT  U/L(≤100/ >100; ref=≤100) | 415.5(200.3-732.5) | 371.0(255.0-1046.5) | 2.000(0.168-23.775) | 0.583 |  |  |
| Bilirubin  mg/dl(≤1/ >1; ref=≤1) | 1.2(0.6-3.6) | 0.9(0.5-2.8) | 0.508(0.160-1.607) | 0.249 |  |  |
| Platelet count  (≤250/ >250; ref=≤250) | 327(274-419) | 89(80-106) | 0.510(0.136-1.920) | 0.320 |  |  |
| Prothrombin time  (≤110/ >110; ref=≤110) | 97(80-110) | 89(80-106) | 1.500(0.386-5.825) | 0.558 |  |  |
| INR  (≤1/>1; ref=≤1) | 1.02(0.93-1.15) | 1.05(0.93-1.16) | 0.984(0.299-3.243) | 0.979 |  |  |
| Hemoglobin  g/L(≤13/ >13; ref=≤13) | 13(11-13) | 12(11-13) | 1.018(0.284-3.651) | 0.978 |  |  |
| CRP  mg/L(≤10/ >10; ref=≤10) | 11.7(5.0-41.0) | 11.3(5.3-23.4) | 1.444(0.450-4.641) | 0.537 |  |  |
| Operative time  minutes(≤360/ >360; ref=≤360) | 400(360-472) | 380(311-460) | 0.442(0.132-1.477) | 0.185 |  |  |
| Intraop PRBC  (No/Yes;ref=No) | 11(42.3)/15(57.7) | 11(50.0)/11(50.0) | 0.733(0.234-2.297) | 0.594 |  |  |
| Intraop FFP  ((No/Yes(%);ref=No) | 9(34.6)/17(65.4) | 7(31.8)/15(68.2) | 1.134(0.339-3.794) | 0.838 |  |  |
| R1 resection  (R0/R1) (%); ref= R0) | 20(76.9)/6(23.1) | 20(90.9)/2(9.1) | 0.333(0.060-1.854) | 0.210 |  |  |
| MVI  (No/Yes(%);ref=No) | 16(61.5)/9(34.6) | 16(72.7)/5(22.7) | 0.556(0.152-2.027) | 0.373 |  |  |
| LVI  (No/Yes(%);ref=No) | 17(65.4)/7(26.9) | 16(72.7)/5(22.7) | 0.759(0.200-2.885) | 0.686 |  |  |
| Tumor grading  ((G1/G2)/( G3/G4) (%);ref= G1/G2) | 18(69.2)/6(23.1) | 18(81.8)/3(13.6) | 0.500(0.108-2.314) | 0.375 |  |  |
| pN category  (N0/N1(%);ref=N0) | 8(30.8)/18(69.2) | 14(63.6)/8(36.4) | 0.254(0.076-0.846) | 0.026 | 0.254(0.076-0.846) | 0.026 |
| ICU time  days(≤1＞1(%),ref=1） | 12(46.2)/14(53.8) | 11(50.0)/11(50.0) | 0.857(0.275-2.672) | 0.790 |  |  |
| Hospitalization  days(≤14/>14(%); ref=≤14) | 20(15-36) | 24(14-44) | 0.800(0.216-2.961) | 0.735 |  |  |
| Adjuvant therapy  (No/Yes(%);ref=No) | 20(76.9)/6(23.0) | 17(77.3)/5(22.7) | 0.980(0.254-3.787) | 0.977 |  |  |
| **Sarcopenia** | **No(n=21)** | **Yes(n=27)** | **OR(95%Cl)** | **p=** | **OR(95%Cl)** | **p=** |
| Sex  (male/female(%); ref=male) | 19(90.5)/2(9.5) | 10(37.0)/17(63.0) | 16.150(3.092-84.361) | 0.001 | 5.523(0.410-62.548) | 0.190 |
| Age  (≤65/ >65 years; ref=≤65) | 64(53-70) | 69(58-74) | 1.939(0.610-6.162) | 0.261 |  |  |
| PVE  (No/Yes(%);ref=No) | 9(42.9)/12(57.1) | 19(70.4)/8(29.6) | 0.316(0.096-1.044) | 0.059 |  |  |
| ASA  ((I/II)/(III/IV) (%);ref= I/II) | 10(47.6)/11(52.4) | 14(51.9)/13(48.1) | 0.844(0.270-2.644) | 0.771 |  |  |
| Neoadjuvant therapy  ((No/Yes(%);ref=No) | 20(95.2)/1(4.8) | 25(92.6)/2(7.4) | 1.600(0.135-18.943) | 0.709 |  |  |
| AST  U/L(≤40/ >40; ref=≤40) | 48(38.0-156.5) | 70.0(28.0-151.0) | 1.781(0.539-5.888) | 0.344 |  |  |
| ALT  U/L(≤40/ >40; ref=≤40) | 48.0(37.5-156.5) | 54.0(37.0-95.0) | 0.433(0.070-2.669) | 0.367 |  |  |
| GGT  U/L(≤100/ >100; ref=≤100) | 349.0(198.0-705.0) | 530.0(246.3-982.0) | 0.667(0.056-7.937) | 0.748 |  |  |
| Bilirubin  mg/dl(≤1/ >1; ref=≤1) | 1.0(0.5-2.8) | 1.2(0.5-3.6) | 1.185(0.378-3.710) | 0.771 |  |  |
| Platelet count  (≤250/ >250; ref=≤250) | 294(247-442) | 354(247-388) | 0.893(0.238-3.351) | 0.867 |  |  |
| Prothrombin time  (≤110/ >110; ref=≤110) | 95(79-111) | 89(81-109) | 0.857(0.220-3.337) | 0.824 |  |  |
| INR  (≤1/>1; ref=≤1) | 1.03(0.92-1.16) | 1.07(0.93-1.14) | 1.333(0.402-4.428) | 0.639 |  |  |
| Hemoglobin  g/L(≤13/ >13; ref=≤13) | 294(347-442) | 354(247-388) | 0.875(0.243-3.146) | 0.838 |  |  |
| CRP  mg/L(≤10/ >10; ref=≤10) | 8.4(5.0-35.0) | 15.6(6.3-41.0) | 1.667(0.515-5.399) | 0.394 |  |  |
| Operative time  minutes(≤360/ >360; ref=≤360) | 370(328-478) | 390(360-450) | 2.597(0.771-8.747) | 0.123 |  |  |
| Intraop PRBC  (No/Yes;ref=No) | 12(57.1)/9(42.9) | 7(25.9)/20(74.1) | 2.267(0.707-7.266) | 0.169 |  |  |
| Intraop FFP  ((No/Yes(%);ref=No) | 9(42.9)/12(57.1) | 19(90.5)/2(9.5) | 2.143(0.633-7.256) | 0.221 |  |  |
| R1 resection  (R0/R1) (%); ref= R0) | 17(81.0)/4(19.0) | 23(85.2)/4(14.8) | 0.739(0.161-3.383) | 0.697 |  |  |
| MVI  (No/Yes(%);ref=No) | 12(57.1)/8(38.1) | 20(74.1)/6(22.2) | 0.450(0.125-1.615) | 0.221 |  |  |
| LVI  (No/Yes(%);ref=No) | 14(66.7)/6(28.6) | 19(70.4)/6(22.2) | 0.737(0.196-2.774) | 0.652 |  |  |
| Tumor grading  ((G1/G2)/( G3/G4) (%);ref= G1/G2) | 16(76.2)/3(14.3) | 20(74.1)/6(22.2) | 1.600(0.345-7.418) | 0.548 |  |  |
| pN category  (N0/N1(%);ref=N0) | 9(42.9)/12(57.1) | 13(48.1)/14(51.9) | 0.808 (0.256-2.545) | 0.715 |  |  |
| ICU time  days(≤1＞1(%),ref=1） | 11(52.4)/10(47.6) | 12(44.4)/15(55.6) | 1.375(0.438-4.318) | 0.585 |  |  |
| Hospitalization  days(≤14/>14(%); ref=≤14) | 23(15-40) | 23(13-36) | 0.559(0.142-2.193) | 0.404 |  |  |
| Adjuvant therapy  (No/Yes(%);ref=No) | 16(76.2)/5(23.8) | 21(77.8)/6(22.2) | 0.914(0.236-3.539） | 0.897 |  |  |
| Normal CD68 grp  (grouped by median, ref=low expression) | 7183.65(2857.17-23921.77) | 16799.60(7070.04-26386.84) | 5.25(1.093-7.437) | 0.038 | 13.581(1.572-117.294) | 0.018 |
| Normal CD8 TIM-3 grp  (grouped by median, ref=low expression) | 84.25(0-454.03) | 1.43 (0-881.64) | 13.333(1.434-123.989） | 0.023 | 20.324(1.579-261.608) | 0.021 |
| **Myosteatosis** | **No(n=25)** | **Yes(n=23)** | **OR(95%Cl)** | **p=** | **OR(95%Cl)** | **p=** |
| Sex  (male/female(%); ref=male) | 16(64.0)/9(36.0) | 13(56.5)/10(43.5) | 1.368(0.429-4.364) | 0.597 |  |  |
| Age  (≤65/ >65 years; ref=≤65) | 64(55-72) | 68(58-74) | 2.812(0.870-9.091) | 0.084 |  |  |
| PVE  (No/Yes(%);ref=No) | 14(56.0)/11(44.0) | 14(60.9)/9(39.1) | 0.818(0.259-2.587) | 0.733 |  |  |
| ASA  ((I/II)/(III/IV) (%);ref= I/II) | 13(52.0)/12(48.0) | 11(47.8)/12(52.2) | 1.182(0.380-3.672) | 0.773 |  |  |
| Neoadjuvant therapy  ((No/Yes(%);ref=No) | 24(96.0)/1(4.0) | 21(91.3)/2(8.7) | 2.286(0.193-27.046) | 0.512 |  |  |
| AST  U/L(≤40/ >40; ref=≤40) | 53.0(37.0-95.0) | 50.0(37.5-86.5) | 1.055(0.323-3.448) | 0.930 |  |  |
| ALT  U/L(≤40/ >40; ref=≤40) | 84.5(34.0-243.3) | 83.5(50.0-164.5) | 1.286(0.385-4.297) | 0.683 |  |  |
| GGT  U/L(≤100/ >100; ref=≤100) | 412.0(211.3-974.8) | 380.0(207.5-719.0) | 0.500 (0.042-5.944) | 0.583 |  |  |
| Bilirubin  mg/dl(≤1/ >1; ref=≤1) | 1.3(0.7-2.8) | 0.6(0.5-3.5) | 0.429(0.135-1.365) | 0.152 |  |  |
| Platelet count  (≤250/ >250; ref=≤250) | 306(230-451) | 354(270-386) | 1.400(0.374-5.244) | 0.618 |  |  |
| Prothrombin time  (≤110/ >110; ref=≤110) | 94(82-113) | 89(77-108) | 0.833(0.215-3.230) | 0.792 |  |  |
| INR  (≤1/>1; ref=≤1) | 10(37.0) | 7(33.3) | 1.125(0.342-3.703) | 0.846 |  |  |
| Hemoglobin  g/L(≤13/ >13; ref=≤13) | 12.2(11.0-13.2) | 11.9(10.8-13.1) | 0.908(0.253-3.251) | 0.882 |  |  |
| CRP  mg/L(≤10/ >10; ref=≤10) | 10.0(6.7-27.5) | 15.9(3.8-52.6) | 2.022(0.624-6.549) | 0.240 |  |  |
| Operative time  minutes(≤360/ >360; ref=≤360) | 370(330-425) | 415(385-473) | 2.226(0.657-7.545) | 0.199 |  |  |
| Intraop PRBC  (No/Yes;ref=No) | 13(52.0)/12(48.0) | 9(39.1)/14(60.9) | 1.610(0.494-5.246) | 0.430 |  |  |
| Intraop FFP  ((No/Yes(%);ref=No) | 10(40.0)/15(60.0) | 6(26.1)/17(73.9) | 1.889(0.554-6.445) | 0.310 |  |  |
| R1 resection  (R0/R1) (%); ref= R0) | 22(88.0)/3(12.0) | 18(78.3)/5(21.7) | 2.037(0.428-9.704) | 0.372 |  |  |
| MVI  (No/Yes(%);ref=No) | 16(64.0)/8(32.0) | 16(69.6)/6(26.0) | 0.750(0.212-2.658) | 0.656 |  |  |
| LVI  (No/Yes(%);ref=No) | 17(68.0)/7(28.0) | 16(69.6)/5(21.7) | 0.759(0.200-2.885) | 0.686 |  |  |
| Tumor grading  ((G1/G2)/( G3/G4) (%);ref= G1/G2) | 17(68.0)/5(20.0) | 19(82.6)/4(17.4) | 0.716(0.165-3.109) | 0.655 |  |  |
| pN category  (N0/N1(%);ref=N0) | 13(52.0)/12(48.0) | 9(39.1)/14(60.9) | 1.685(0.535-5.309) | 0.373 |  |  |
| ICU time  days(≤1＞1(%),ref=1） | 12(48.0)/13(53.0) | 11(47.8)/12(52.2) | 1.007(0.324-3.128) | 0.990 |  |  |
| Hospitalization  days(≤14/>14(%); ref=≤14) | 23(14-38) | 23(14-39) | 1.400(0.374-5.244) | 0.618 |  |  |
| Adjuvant therapy  (No/Yes(%);ref=No) | 19(76.0)/6(24.0) | 18(78.3)/5(21.7) | 0.880(0.228-3.395) | 0.852 |  |  |
| Tumor CD68 PD-L2 grp  (grouped by median, ref=low expression) | 527.67 (4.27-1281.34) | 925.33 (23.90-1902.77) | 4.375(1.210-15.812) | 0.024 | 8.509(1.570-46.118) | 0.013 |
| Tumor CD4 grp  (grouped by median, ref=low expression) | 3059.79 (762.03-3749.18) | 2481.31 (359.70-4439.26) | 0.215(0.058-0.806) | 0.023 | 0.118(0.022-0.637) | 0.013 |
| **VFA** | **≤100(n=21)** | **＞100(n=27)** | **OR(95%Cl)** | **p=** | **OR(95%Cl)** | **p=** |
| Sex  (male/female(%); ref=male) | 11(52.4)/10(47.6) | 18(66.7)/9(33.3) | 0.550(0.170-1.776) | 0.317 |  |  |
| Age  (≤65/ >65 years; ref=≤65) | 64(55-71) | 68(57-75) | 1.375(0.438-4.318) | 0.585 |  |  |
| PVE  (No/Yes(%);ref=No) | 15(71.4)/6(28.6) | 13(48.1)/14(51.9) | 2.692(0.802-9.037) | 0.109 |  |  |
| ASA  ((I/II)/(III/IV) (%);ref= I/II) | 11(52.4)/10(47.6) | 13(48.1)/14(51.9) | 1.185(0.378-3.710) | 0.771 |  |  |
| Neoadjuvant therapy  ((No/Yes(%);ref=No) | 19(90.5)/2 (9.5) | 26(96.3)/1(3.7) | 0.365(0.031-4.330) | 0.425 |  |  |
| AST  U/L(≤40/ >40; ref=≤40) | 48.0(32.0-76.0) | 54.0(39.0-131.0) | 1.231(0.374-4.045) | 0.732 |  |  |
| ALT  U/L(≤40/ >40; ref=≤40) | 83.5(55.5-144.5) | 84.5(39.5-121.0) | 0.462(0.437-4.889) | 0.538 |  |  |
| GGT  U/L(≤100/ >100; ref=≤100) | 451.0(208.0-872.0) | 360.0(195.3-834.3) | 0.667(0.056-7.937) | 0.748 |  |  |
| Bilirubin  mg/dl(≤1/ >1; ref=≤1) | 1.3(0.7-3.2) | 0.9(0.5-2.9) | 0.423(0.132-1.361) | 0.149 |  |  |
| Platelet count  (≤250/ >250; ref=≤250) | 354(276-474) | 300(230-420) | 0.333(0.077-1.437) | 0.140 |  |  |
| Prothrombin time  (≤110/ >110; ref=≤110) | 96(79-115) | 89(80-100) | 0.530(0.136-2.072) | 0.362 |  |  |
| INR  (≤1/>1; ref=≤1) | 1.03(0.92-1.16) | 1.05(0.96-1.10) | 1.943(0.581-6.501) | 0.281 |  |  |
| Hemoglobin  g/L(≤13/ >13; ref=≤13) | 12(10-13) | 12(11-13) | 0.369(0.100-1.370) | 0.136 |  |  |
| CRP  mg/L(≤10/ >10; ref=≤10) | 12.8(5.4-51.5) | 11.3(5.0-27.2) | 1.167(0.363-3.749) | 0.796 |  |  |
| Operative time  minutes(≤360/ >360; ref=≤360) | 390(365-450) | 385(330-473) | 0.391(0.111-1.375) | 0.143 |  |  |
| Intraop PRBC  (No/Yes;ref=No) | 10(47.6)/11(52.4) | 12(44.4)/15(55.6) | 1.136(0.362-3.569) | 0.827 |  |  |
| Intraop FFP  ((No/Yes(%);ref=No) | 7(33.3)/14(66.7) | 9(33.3)/18(66.7) | 1(0.298-3.353) | 1 |  |  |
| R1 resection  (R0/R1) (%); ref= R0) | 18(85.7)/3(14.3) | 22(81.5)/5(18.5) | 1.364(0.286-6.496) | 0.697 |  |  |
| MVI  (No/Yes(%);ref=No) | 15(71.4)/5(23.8) | 17(63.0)/9(33.3) | 1.588(0.435-5.799) | 0.484 |  |  |
| LVI  (No/Yes(%);ref=No) | 18(85.7)/3(14.3) | 15(55.6)/9 (33.3) | 3.600(0.823-15.742) | 0.089 |  |  |
| Tumor grading  ((G1/G2)/( G3/G4) (%);ref= G1/G2) | 15(71.4)/5(23.8) | 21(77.8)/4(14.8) | 0.571(0.131-2.491) | 0.456 |  |  |
| pN category  (N0/N1(%);ref=N0) | 10(47.6)/11(52.4) | 12(44.4)/15(55.6) | 1.136(0.362-3.569) | 0.827 |  |  |
| ICU time  days(≤1＞1(%),ref=1） | 9(42.9)/12(57.1) | 14(51.9)/13(48.1) | 0.696(0.221-2.194) | 0.537 |  |  |
| Hospitalization  days(≤14/>14(%); ref=≤14) | 19(12-36) | 24(15-45) | 2.200(0.582-8.309) | 0.245 |  |  |
| Adjuvant therapy  (No/Yes(%);ref=No) | 15(71.4)/6(28.6) | 22(81.5)/5 (18.5) | 0.568(0.146-2.205) | 0.414 |  |  |
| Tumor CD4 ICOS TIGIT CTLA-4 grp  (grouped by median, ref=low expression) | 0 (0-20.32) | 8.27 (0-28.75) | 0.25(0.066-0.946) | 0.041 | 8.530(0.241-302.501) | 0.239 |
| Tumor CD4 TIGIT grp  (grouped by median, ref=low expression) | 58.25 (2.80-204.79) | 46.30 (5.89-126.62) | 0.250(0.066-0.946) | 0.041 | 0.509(0.083-3.130) | 0.466 |
| Tumor CD8 ICOS TIGIT CTLA-4 grp  (grouped by median, ref=low expression) | 7.88 (0-128.20) | 7.44 (0-49.75) | 0.25(0.066-0.946) | 0.041 | n.a. | 1 |
| Tumor CD8 TIGIT grp  (grouped by median, ref=low expression) | 153.84(38.09-573.73) | 153.79 (12.29-333.11) | 0.25(0.066-0.946) | 0.041 | 1.562(0.053-46.293) | 0.797 |
| Tumor CD8 ICOS CTLA-4 grp  (grouped by median, ref=low expression) | 14.26 (0-960.67) | 9.10 (0-157.62) | 0.25(0.066-0.946) | 0.041 | 4.977(0.167-148.397) | 0.354 |
| Tumor CD8 TIGIT CTLA-4 grp  (grouped by median, ref=low expression) | 18.29 (0-40.595) | 17.38 (1.27-150.57) | 0.25(0.066-0.946) | 0.041 | 0.276(0.048-1.584) | 0.149 |
| Normal CD4 ICOS TIGIT grp  (grouped by median, ref=low expression) | 0 (0-80.02) | 0(0-0) | 0.179（0.037-0.863） | 0.032 | 0.153(0.028-0.833) | 0.030 |
| **Sarcopenic obesity** | **No(n=41)** | **Yes(n=7)** | **OR(95%Cl)** | **p=** | **OR(95%Cl)** | **p=** |
| Sex  (male/female(%); ref=male) | 23(56.1)/18(43.9) | 6(85.7)/1(14.3) | 0.213(0.023-1.932) | 0.169 |  |  |
| Age  (≤65/ >65 years; ref=≤65) | 67.0(55.0-72.8) | 66.0(58.0-73.5) | 0.648(0.128-3.266) | 0.599 |  |  |
| PVE  (No/Yes(%);ref=No) | 22(53.7)/19(46.3) | 6(85.7)/1(14.3) | 0.193(0.021-1.749) | 0.144 |  |  |
| ASA  ((I/II)/(III/IV) (%);ref= I/II) | 21(51.2)/20(48.8) | 3(42.9)/4(57.1) | 1.400(0.278-7.056) | 0.683 |  |  |
| Neoadjuvant therapy  ((No/Yes(%);ref=No) | 40(97.6)/1(2.4) | 5(71.4)/2(28.6) | 2.714(0.216-34.149) | 0.440 |  |  |
| AST  U/L(≤40/ >40; ref=≤40) | 53.5(37.0-94.0) | 48.5(40.5-183.0) | 0.691(0.135-3.529) | 0.657 |  |  |
| ALT  U/L(≤40/ >40; ref=≤40) | 94.0(60.0-199.0) | 28.0(20.5-290.5) | 0.429(0.092-2.003) | 0.282 |  |  |
| GGT  U/L(≤100/ >100; ref=≤100) | 380.0(229.5-767.0) | 423.0(159.3-1577.5) | n.a | 0.999 |  |  |
| Bilirubin  mg/dl(≤1/ >1; ref=≤1) | 1.2(0.5-3.6) | 0.7(0.4-1.7) | 0.714(0.142-3.600) | 0.683 |  |  |
| Platelet count  (≤250/ >250; ref=≤250) | 317(265-420) | 367(234-524) | 0.375(0.071-1.991) | 0.250 |  |  |
| Prothrombin time  (≤110/ >110; ref=≤110) | 93(79-109) | 91(84-111) | 0.500(0.054-4.672) | 0.543 |  |  |
| INR  (≤1/>1; ref=≤1) | 1.03(0.93-1.16) | 1.06(0.95-1.13) | 4.000(0.439-36.444) | 0.219 |  |  |
| Hemoglobin  g/L(≤13/ >13; ref=≤13) | 12(11-13) | 12(11-13) | 1.091(0.184-6.465) | 0.924 |  |  |
| CRP  mg/L(≤10/ >10; ref=≤10) | 10.5(5.8-38.2) | 17.1(3.7-23.4) | 0.643(0.127-3.261) | 0.594 |  |  |
| Operative time  minutes(≤360/ >360; ref=≤360) | 388(341-450) | 420(365-472) | 1.442(0.248-8.372) | 0.683 |  |  |
| Intraop PRBC  (No/Yes;ref=No) | 18(43.9)/23(56.1) | 4(57.1)/3(42.9) | 0.587(0.116-2.963) | 0.519 |  |  |
| Intraop FFP  ((No/Yes(%);ref=No) | 13(31.7)/28(68.3) | 3(42.9)/4(57.1) | 0.619(0.121-3.176) | 0.565 |  |  |
| R1 resection  (R0/R1) (%); ref= R0) | 33(80.5)/8(19.5) | 7(100)/0(0) | 0(0-0) | 0.999 |  |  |
| MVI  (No/Yes(%);ref=No) | 26(63.4)/13(31.7) | 6(85.7)/1(14.3) | 0.333(0.036-3.067) | 0.332 |  |  |
| LVI  (No/Yes(%);ref=No) | 27(65.9)/11(26.8) | 6(85.7)/1(14.3) | 0.409(0.044-3.805) | 0.432 |  |  |
| Tumor grading  ((G1/G2)/( G3/G4) (%);ref= G1/G2) | 30(73.2)/8(19.5) | 6(85.7)/1(14.3) | 0.625(0.065-5.966) | 0.683 |  |  |
| pN category  (N0/N1(%);ref=N0) | 17(45.1)/24(58.5) | 5(71.4)/2(28.6) | 0.283(0.049-1.636) | 0.159 |  |  |
| ICU time  days(≤1＞1(%),ref=1） | 18(43.9)/23(56.1) | 5(71.4)/2(28.5) | 0.313 (0.054-1.805) | 0.194 |  |  |
| Hospitalization  days(≤14/>14(%); ref=≤14) | 24(15-38) | 15(12-38) | 0.182(0.034-0.982) | 0.047 | 0.153(0.014-1.732) | 0.130 |
| Adjuvant therapy  (No/Yes(%);ref=No) | 33(80.5)/8(19.5) | 4(57.1)/3(42.9) | 3.094(0.574-16.674) | 0.189 |  |  |
| Normal CD8 PD-1 PD-L1 PD-L2 grp  (grouped by median, ref=low expression) | 0 (0-0) | 0 (0-61.17) | 9.200(1.304-64.895） | 0.026 | 15.884(1.447-174.360） | 0.024 |

Note: The multiplex data were divided into high and low expression groups based on the median.

Abbreviations: ALT, alanine aminotransferase;ASA,American Society of Anesthesiologists;AST, aspartate aminotransferase; BMI, body mass index; CRP, C-­reactive protein; F, female; FFP,Fresh frozen plasma; GGT,gamma-glutamyl transferase; HR, hazard ratio; iCCA, intrahepatic cholangiocarcinoma; ICU, intensive care unit; INR, international normalized ratio; LVI, lymph vascular invasion; M, male; MVI, microvascular invasion; PRBC, packed red blood cells; PVE, portal vein embolization; RFS, recurrence free survival. Variables displaying a p value < 0.05 in the univariate analysis were transferred into a multivariable logistic regression model.

Note: multiplex date(×10^-5^).
