# Supplementary material for: Body Composition in Cholangiocarcinoma Affects Immune Cell Populations in the Tumor and Normal Liver Parenchyma
Source: J Clin Exp Hepatol. 2024 Nov 26;15(2):102460. doi: 10.1016/j.jceh.2024.102460 (PMC11697564; doi:10.1016/j.jceh.2024.102460)
Supplement: Multimedia component 5 [file mmc5.docx]

Supplementary Table S3 Comparison of CD8+ T Cell Density (High vs Low) in iCCA Patients

| **Variables** | CD8+ T cells | | |
| --- | --- | --- | --- |
|  | Low(n=24) | High(n=24) | P Value |
| **Demographics** |  |  |  |
| Sex,m/f(%) | 8(33.3)/16(66.7) | 15(62.5)/9(37.5) | 0.149 |
| Age(years) | 66(58-72) | 67(53-73) | 0.364 |
| Portal vein embolization, n (%) | 2(8.3) | 1(4.2) |  |
| ASA,n(%) |  |  | 0.577 |
| I | 0(0) | 1(4.2) |  |
| II | 14(58.3) | 9(37.5) |  |
| III | 8(33.3) | 12(50.0) |  |
| IV | 2(8.3) | 2(8.3) |  |
| Preoperative Chemotherapy, n (%) | 2(8.3) | 1(4.2) | 1.0 |
| Clinical chemistry | | | |
| AST (U/l) | 38.0(26.0-47.0) | 39.5(26.0-62.5) | 0.804 |
| ALT (U/l) | 24.0(17.0-73.5) | 32.0(21.5-68.0) | 0.483 |
| GGT (U/l) | 85(67.0-289.0) | 220.0(77.0-501.0) | 0.245 |
| Total bilirubin (mg/dl) | 0.4(0.4-0.8) | 0.6(0.5-1.4) | 0.080 |
| Hemoglobin (g/dl) | 12.9(11.9-14.6) | 13.3(12.3-14.1) | 0.433 |
| Platelet count (/nl) | () | () |  |
| INR | 0.9(0.9-1.0) | 1.0(0.9-1.1) | 0.036 |
| Prothrombin time (%) | 100.0(95.0-113.3) | 98.0(84.0-102.0) | 0.042 |
| CRP (mg/l) | 11.0(3.3-38.5) | 9.5(4.9-22.0) | 0.644 |
| **Operative Data** |  |  |  |
| Operative time (minutes) | 285(230-353) | 290(222-354) | 0.718 |
| Intraoperative PRBC, n (%) | 0(0-2) | 0(0-2) | 0.853 |
| Intraoperative FFP, n (%) | 0(0-4) | 0(0-5) | 0.627 |
| **Pathological examination** |  |  |  |
| R1 resection, n (%) | 2(8.3) | 2(8.3) | 0.214 |
| pN category, n (%) |  |  | 0.460 |
| N0 | 15(62.5) | 16(66.7) |  |
| N1 | 9(37.5) | 6(25.0) |  |
| Tumor grading, n (%) |  |  | 0.425 |
| G1 | 8(33.3) | 7(29.2) |  |
| G2 | 11(45.8) | 11(45.8) |  |
| G3 | 3(12.5) | 3(12.5) |  |
| G4 | 2(8.3) | 3(12.5) |  |
| MVI, n (%) | 0(0) | 1(4.2) | 1.0 |
| LVI, n (%) | 7(29.2) | 2(8.3) | 0.117 |
| pT category n (%) |  |  | 0.357 |
| 1 | 8(33.3) | 4(29.2) |  |
| 2 | 11(45.8) | 11(45.8) |  |
| 3 | 3(12.5) | 3(12.5) |  |
| 4 | 2(8.3) | 3(12.5) |  |
| **Postoperative Data** |  |  |  |
| Intensive care, days | 1(1-3) | 1(1-1) | 0.105 |
| Hospitalization, days | 15(10-26) | 13(8-23) | 0.311 |
| **Oncologic Data** |  |  |  |
| Adjuvant chemotherapy, n (%) | 7(29.2) | 5(20.8) | 0.505 |
| Recurrence, n (%) | 17(70.8) | 18(75.0) | 0.559 |
| **Body composition** |  |  |  |
| Obesity,n(%) | 13(54.2) | 11(45.8) | 0.564 |
| Visceral fat ,n (%) | 13(54.2) | 13(54.2) | 1.000 |
| Sarcopenia, n (%) | 19(79.2) | 15(62.5) | 0.204 |
| Myosteatosis, n (%) | 16(66.7) | 12(50.0) | 0.242 |
| Sarcopenic obesity, n (%) | 5(20.8) | 4(16.7) | 0.719 |
