# Supplementary material for: Body Composition in Cholangiocarcinoma Affects Immune Cell Populations in the Tumor and Normal Liver Parenchyma
Source: J Clin Exp Hepatol. 2024 Nov 26;15(2):102460. doi: 10.1016/j.jceh.2024.102460 (PMC11697564; doi:10.1016/j.jceh.2024.102460)
Supplement: Multimedia component 6 [file mmc6.docx]

Supplementary Table S4 Comparison of CD8+ T Cell Density (High vs Low) in pCCA Patients

| **Variables** | CD8+ T cells | | |
| --- | --- | --- | --- |
|  | Low(n=24) | High(n=24) | P Value |
| **Demographics** |  |  |  |
| Sex,m/f(%) | 15(62.5)/9(37.5) | 14(58.3)/10(41.7) | 0.768 |
| Age(years) | 65(54-73) | 68(60-73) | 0.375 |
| Portal vein embolization, n (%) | 12(50.0) | 8(33.3) |  |
| ASA,n(%) |  |  | 0.177 |
| I | 0(0) | 2(8.3) |  |
| II | 12(50.0) | 10(41.7) |  |
| III | 12(50.0) | 11(45.8) |  |
| IV | 0(0) | 1(4.2) |  |
| Preoperative Chemotherapy, n (%) | 1(4.2) | 2(8.3) | 0.242 |
| Clinical chemistry | | | |
| AST (U/l) | 48.0(34.8-74.3) | 63.5(38.3-182.3) | 0.180 |
| ALT (U/l) | 84.5(52.5-134.0) | 94.0(39.5-274.0) | 0.696 |
| GGT (U/l) | 352.0(251.0-973.0) | 574.5(188.5-809.0) | 0.658 |
| Total bilirubin (mg/dl) | 1.05(0.5-2.9) | 1.1(0.5-4.0) | 0.853 |
| Hemoglobin (g/dl) | 12.4(11.5-13.1) | 11.7(10.3-13.3) | 0.426 |
| Platelet count (/nl) | () | () |  |
| INR | 1.1(0.9-1.1) | 1.0(0.9-1.2) | 0.580 |
| Prothrombin time (%) | 89.0(76.7-100) | 94.0(81.0-114.0) | 0.426 |
| CRP (mg/l) | 11.0(5.0-37.0) | 11.6(6.6-37.3) | 0.809 |
| **Operative Data** |  |  |  |
| Operative time (minutes) | 393(346-450) | 383(355-474) | 0.926 |
| Intraoperative PRBC, n (%) | 2(0-2) | 0(0-2) | 0.063 |
| Intraoperative FFP, n (%) | 4(3-6) | 3(0-4) | 0.132 |
| **Pathological examination** |  |  |  |
| R1 resection, n (%) | 2(8.3) | 6(25.0) | 0.245 |
| pN category, n (%) |  |  | 1.0 |
| N0 | 11(45.8) | 11(45.8) |  |
| N1 | 13(54.2) | 13(54.2) |  |
| Tumor grading, n (%) |  |  | 0.213 |
| G1 | 0(0) | 0(0) |  |
| G2 | 19(79.2) | 17(70.8) |  |
| G3 | 2(8.3) | 6(25.0) |  |
| G4 | 1(4.2) | () |  |
| MVI, n (%) | 0(0) | 1(8.3) | 0.470 |
| LVI, n (%) | 5(20.8) | 7(29.2) | 0.685 |
| pT category n (%) |  |  | 0.465 |
| 1 | 0(0) | 0(0) |  |
| 2 | 17(70.8) | 12(50.0) |  |
| 3 | 5(20.8) | 7(29.2) |  |
| 4 | 2(8.3) | 5(20.9) |  |
| **Postoperative Data** |  |  |  |
| Intensive care, days | 1(1-2) | 1(1-2) | 0.696 |
| Hospitalization, days | 24(16-41) | 19(12-38) | 0.302 |
| **Oncologic Data** |  |  |  |
| Adjuvant chemotherapy, n (%) | 4(16.7) | 7(29.2) | 0.303 |
| Recurrence, n (%) | 15(62.5) | 17(70.8) | 0.845 |
| **Body composition** |  |  |  |
| Obesity,n(%) | 11(45.8) | 10(41.7) | 0.771 |
| Visceral fat ,n (%) | 14(58.3) | 13(54.2) | 0.771 |
| Sarcopenia, n (%) | 16(66.7) | 11(45.8) | 0.146 |
| Myosteatosis, n (%) | 13(54.2) | 8(33.3) | 0.146 |
| Sarcopenic obesity, n (%) | 5(20.8) | 3(12.5) | 0.439 |
